# Activation of cellular responses by cyclic dinucleotides and *porphyromonas gingivalis* lipopolysaccharide: a proteomic study on gingival fibroblasts

**DOI:** 10.1080/20002297.2024.2431453

**Published:** 2024-12-09

**Authors:** Samira Elmanfi, Kenneth I. Onyedibe, Uma K. Aryal, Eija Könönen, Herman O. Sintim, Ulvi Kahraman Gürsoy

**Affiliations:** a Department of Periodontology, Institute of Dentistry, University of Turku, Turku, Finland; b Department of Chemistry, Purdue University, West Lafayette, USA; c Purdue Institute for Inflammation, Immunology and Infectious Disease and Purdue Institute for Drug Discovery, Purdue University, West Lafayette, USA; d Department of Biomedical Sciences, Mercer University School of Medicine, Macon GA, USA; e Purdue Proteomics Facility, Bindley Bioscience Center, Purdue University, West Lafayette, USA; f Department of Comparative Pathobiology, Purdue University, West Lafayette, USA

**Keywords:** Lipopolysaccharide, *porphyromonas gingivalis*, proteomic analysis, cyclic dinucleotides, human gingival fibroblasts, innate immune response, interferon signaling

## Abstract

**Background:**

Bacterial cyclic dinucleotides (CDNs), cyclic di-guanosine monophosphate (c-di-GMP), and cyclic di-adenosine monophosphate (c-di-AMP) upregulate interferon signaling proteins of human gingival fibroblasts (HGFs). However, the simultaneous effect of bacterial CDNs and lipopolysaccharides (LPS) on the HGF proteome is unknown.

**Aim:**

The aim was to apply an unbiased proteomics approach to evaluate how simultaneous exposure to CDNs and *Porphyromonas gingivalis* (Pg) LPS affect the global proteome of HGFs.

**Methods:**

The proteomic responses of HGFs were examined under three different treatment conditions (c-di-AMP+Pg LPS, c-di-GMP+Pg LPS, and Pg LPS alone) by label-free quantitative mass spectrometry analysis.

**Results:**

Simultaneous exposure to CDNs and Pg LPS significantly upregulated innate immunity-related and interferon signaling-related proteins, such as ubiquitin-like protein ISG15 (ISG15), deoxynucleoside triphosphate triphosphohydrolase (SAMHD1), interferon regulatory factor 9 (IRF-9), interferon-induced GTP-binding protein Mx (MX)1, and MX2. Interferon signaling pathway was the most significantly regulated canonical pathway in both CDN treatment groups.

**Conclusion:**

Simultaneous exposure to CDNs and Pg LPS stimulates the periodontal immune response by activating the anti-microbial cellular responses of HGFs with some notable differences from individual exposures.

## Introduction

Human gingival fibroblasts (HGFs) are predominant cells in healthy periodontal connective tissues [1], being responsible for the homeostasis of connective tissue and renewal of the extracellular matrix [2,3]. Moreover, HGFs recognize and respond to pathogenic microorganisms through their pathogen recognition receptors [4]. Of those, toll-like receptors (TLRs) are significant players in immunity by recognizing and binding to microbe-associated molecular patterns (MAMPs), including lipopolysaccharides (LPS) of the outer membrane of Gram-negative bacteria [4–6]. In HGFs, the recognition of MAMPs by TLRs stimulates an immune response by activating signaling pathways and secreting proinflammatory cytokines, like, tumor necrosis factor (TNF)α, interferonγ, interleukin (IL)-6, IL-8, IL-10, and granulocyte/macrophage colony-stimulating factor [7,8]. In the HGF, these TLR-induced responses mediate major signaling pathways via nuclear factor-κB (NF-κB), mitogen-activated protein kinases (MAPKs), and interferon regulatory factors (IRFs) to produce type 1 interferons and other inflammatory cytokines [9]. LPS of *Porphyromonas gingivalis (Pg)*, a well-known periodontitis-associated bacterium, can induce the expression of IL-6 and IL-8 through activation of nucleotide oligomerization domain-like receptors 1/2 and extracellular signal-regulated kinase 1/2 signaling pathways mediated by NF-κB and TLRs, respectively [10,11]. *P.gingivalis* produces multiple types of LPS to adapt to and survive in various environmental conditions *in vivo*, thereby enhancing its survival and virulence. O-LPS refers to a conventional O-antigen polysaccharide found in most Gram-negative bacteria, while A-LPS is an anionic polysaccharide. Both O-LPS and A-LPS forms are linked to lipid A [12]. Furthermore, *Pg* LPS activates many second-messenger systems of gingival fibroblasts; among those are tyrosine kinases, monocyte chemoattractant protein-1, IL-1 receptor-associated kinase, and activating protein-1 [13].

Bacterial cyclic dinucleotides (CDNs), cyclic di-guanosine monophosphate (c-di-GMP), and cyclic di-adenosine monophosphate (c-di-AMP) are intracellular signaling second messenger systems, which also act as MAMPs [14–16].

C-di-AMP, mainly produced by Gram-positive bacteria, regulates various bacterial cellular processes, such as cell wall metabolism, maintenance of DNA integrity, cell wall homeostasis, biofilm formation, bacterial growth, and antibiotic resistance [17]. While, c-di-GMP, which is mainly expressed by Gram-negative bacteria, regulates bacterial motility, virulence, stress survival, biofilm formation, and differentiation [18,19]. Literature supports the finding that Gram-negative oral bacteria, in particular *Fusobacterium nucleatum*, *Selenomonas sputigena*, *Porphyromonas gingivalis*, *Treponema denticola*, and *Selenomonas noxia*, can synthesise c-di-GMP as well [20–22]. Indeed, these findings support the fact that periodontitis-associated biofilms carry multiple bacteria that can produce both LPS and CDN simultaneously.

Pathogenic stimuli lead to the secretion of bacterial CDNs into the cytosol, which binds the endoplasmic reticulum (ER)-associated stimulator of interferon genes protein (STING). The latter, in turn, activates the TANK-binding kinase 1 (TBK1)-IRF3 pathway and produces type 1 interferons and a robust innate immune response [23].

Profiling how bacterial-derived CDNs affect immune and resident cells of the periodontium has been in the focus of our research [24–27]. We recently demonstrated that bacterial CDNs can stimulate interferon signaling and innate immune responses in HGFs [24] and that CDNs can regulate other critical non-inflammatory processes such as necroptosis signaling, protein ubiquitination, EIF2 signaling, and nucleotide excision repair pathways in HGFs [24]. It is highly likely that during infection, the host would be exposed to bacterial LPS and CDNs, as well as other MAMPs. Indeed, in the context of periodontitis, various MAMPs simultaneously activate different signaling networks. Yet, only a few studies address how the host integrates these signaling networks to shape its final response. Thus, our goal is profiling the host response to multiple MAMPs, which potentially reveal hierarchies or synergies of various MAMPs-related signaling networks. In our previous study, we demonstrated cellular responses of HGFs against simultaneous exposure to *Pg* LPS, c-di-AMP, and c-di-GMP [25]. Nevertheless, this study focused only on selected interleukins and metalloproteinase responses rather than giving a general overview of HGF cellular responses against these MAMPs. In the present study, using an unbiased global proteomics approach, our aim was to examine the effects of simultaneous exposures of *Pg* LPS and bacterial derived CDNs on gingival fibroblasts’ proteome response.

## Materials and methods

### Cell culture

Gingival fibroblasts used in this study were originally isolated from a patient undergoing tooth extraction at the Institute of Dentistry, University of Turku [28]. The patient gave informed consent before tooth extraction. The Ethics Committee of the Hospital District of South-West Finland and the Ethical Committee of the Dentistry, University of Helsinki approved the experimental protocol (Permission date: 19 November 2002, number of the study case: §262). HGFs were cultured in Dulbecco’s modified eagle medium (DMED) with 10% fetal bovine serum (Gibco BRL, Life Technologies), antibiotics (100 IU/mL penicillin and 100 µg/mL streptomycin), and 1% non-essential amino acid (Gibco BRL, Life Technologies), at 37 ◦C and 5% CO_2_. Culture media were changed three times per week; the cells were passaged at 80–90% confluence.

### Synthesis of cyclic dinucleotides

c-di-GMP and c-di-AMP, commercially available from various vendors but expensive, were prepared using the protocol described by Gaffney et al. [29,30]. Briefly, adenosine and guanosine phosphoramidites were coupled to form linear dinucleotide and cyclized to form the cyclic dinucleotide in a one-flask operation, following the established protocol [29].

### Stock preparation of pg LPS

Ultrapure LPS of *Pg* (1 mg) (Invivogen, San Diego, USA) was dissolved in 1 mL of endotoxin-free water to prepare the stock solution (1 mg/ml).

### Incubation of gingival fibroblasts with cyclic dinucleotides

HGFs (7X10^5^/petri dish) were incubated in 3 mL DMEM at 37°C and 5% CO_2_ for 24 h. Phosphate Buffered Saline (PBS) was used to wash the cells. The fibroblasts were incubated at 37°C and 5% CO_2_ for 24 h with fresh media containing 100 µM of c-di-GMP or c-di-AMP with *Pg* LPS (1 µg/mL). The control cells were not incubated with CDNs or *Pg* LPS. After collecting the media, cells were trypsinized by trypsin, and the pellets were collected for proteomics analysis.

### Proteomics analysis

Proteomics analysis was performed at the Purdue Proteomics Facility, as described previously [24,31–33]. Briefly, cells were re-suspended in 100 mm ammonium bicarbonate (ABC) and subjected to high-pressure lysis using a barocycler (Pressure Bioscience Inc., Easton, MS, USA). Protein concentration was determined by bicinchoninic acid (BCA) assay (Pierce Chemical Co., Rockford, IL, USA). Following the BCA assay, cell lysates containing 100 µg protein (equivalent volume), were acetone precipitated, and precipitated protein pellets were reconstituted in 8 M urea, reduced and alkylated prior to trypsin/Lys-C digestion, as described previously [33,34]. LC-MS/MS data were collected on a Thermo Q Exactive Orbitrap HF mass spectrometer and with a Dionex UltiMate 3000 hPLC system using a 120 min LC gradient. One (1) μg of digested peptides were loaded to the trap column (200 μm ID × 5 mm) packed with 5 μm 100 Å PepMap C18 medium and then separated on an Acclaim PepMap 100 Å analytical column (×75 μm ID × 50 cm) packed with 2 μm 100 Å C18 column (Thermo Fisher Scientific, Waltham, MA, USA). Mobile phase A contained 0.1% formic acid (FA) in water, and phase B was 0.1% FA in 80% acetonitrile. The column temperature was maintained at 50°C. The mass spectrometer was operated using a standard data-dependent MS/MS scan method. The full scan MS spectra were collected with a Top20 method for MS/MS fragmentation in the 350–1600 m/z with a maximum injection time of 100 m/s and a resolution of 120 000 at 200 m/z. High-energy C-trap dissociation with the normalized collision energy of 27 eV was used to fragment the precursor. Resolution of MS/MS scans was acquired at 15 000 at m/z 200. Instrument optimization and calibration was carried out at the start of the experiment and then every 72 h. The performance of the instrument was monitored using Hela digest (Pierce) at the start of the experiment and after calibration. To exclude repeated scanning of identical peptides, the dynamic exclusion was set at 20 s. MaxQuant (version 1.6.3.3) and Uniprot human protein database were used for protein identification and label-free quantitation (LFQ) [35]. LC-MS/MS data were collected for three biological replicates per treatment group. The plotting of Venn diagrams was done by the Venny software (Venny. 2.1), and the initial bioinformatics analysis was done using the Perseus software [36]. Only proteins identified in at least two out of the three treatment replicates and with at least two MS/MS counts and LFQ intensity > 0 were analyzed further as previously described [24]. Briefly, LFQ intensities were Log2 transformed, filtered and a student’s t-test with 5% permutation-based false discovery rate (FDR) was used except where otherwise mentioned. Partial least squares-discriminant analysis (PLS-DA) was done by R package of the MetaboAnalyst software Version 5.0. Volcano plots and principal component analysis (PCA) plots were generated in the OriginPro 2020 software (OriginLab, MA). Pathway enrichment and graphics were carried out using the IPA functional network core analysis (QIAGEN Inc., https://www.qiagenbioinformatics.com/products/ingenuity-pathway-analysis) Proteins detected in at least two biological replicates in each of the treatment groups but not found in the control group as well as proteins with Log2 fold change ≥ 0.5 (*p* ≤ 0.05) were considered significantly upregulated by the respective treatment.

## Results

### Differential expression of proteins in human gingival fibroblasts following three treatment conditions

**Figure 1.**
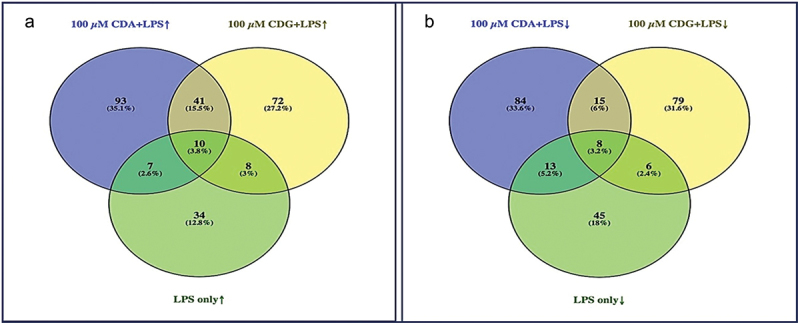
Venn diagram showing (a) upregulated proteins and (b) downregulated proteins (number and percentages) identified in the 100 µm of c-di-AMP+*Pg* LPS, 100 µm c-di-GMP+*Pg* LPS, and *pg* LPS treated gingival fibroblasts. CDA+LPS = c-di-AMP+*Pg* LPS; CDG+ LPS = c-di-GMP+*Pg* LPS; LPS =*Pg* LPS.

In the current work, we aimed to study the differences in protein expression profiles of HGFs following three treatment conditions (c-di-AMP+*Pg* LPS (CDA+LPS), c-di-GMP+*Pg* LPS (CDG+LPS), and *Pg* LPS only (LPS)). As presented in Figure 1a,b the number of upregulated and downregulated proteins differed between the three treatment groups. For c-di-AMP+*Pg* LPS and c-di-GMP+*Pg* LPS, the numbers of upregulated proteins were 93 (35.1%) and 72 (27.2%), respectively, while only 34 (12.8%) proteins were upregulated by *Pg* LPS alone (Figure 1a). Likewise, c-di-AMP+*Pg* LPS downregulated 84 (33.6%) proteins, c-di-GMP+*Pg* LPS 79 (31.6%) proteins, and *Pg* LPS alone 45 (18%) proteins (Figure 1b). For c-di-AMP+*Pg* LPS and c-di-GMP+*Pg* LPS, the number of common upregulated (Figure 1a) and downregulated (Figure 1b) proteins were 41 (15.5%) and 15 (6%), respectively.

**Figure 2.**
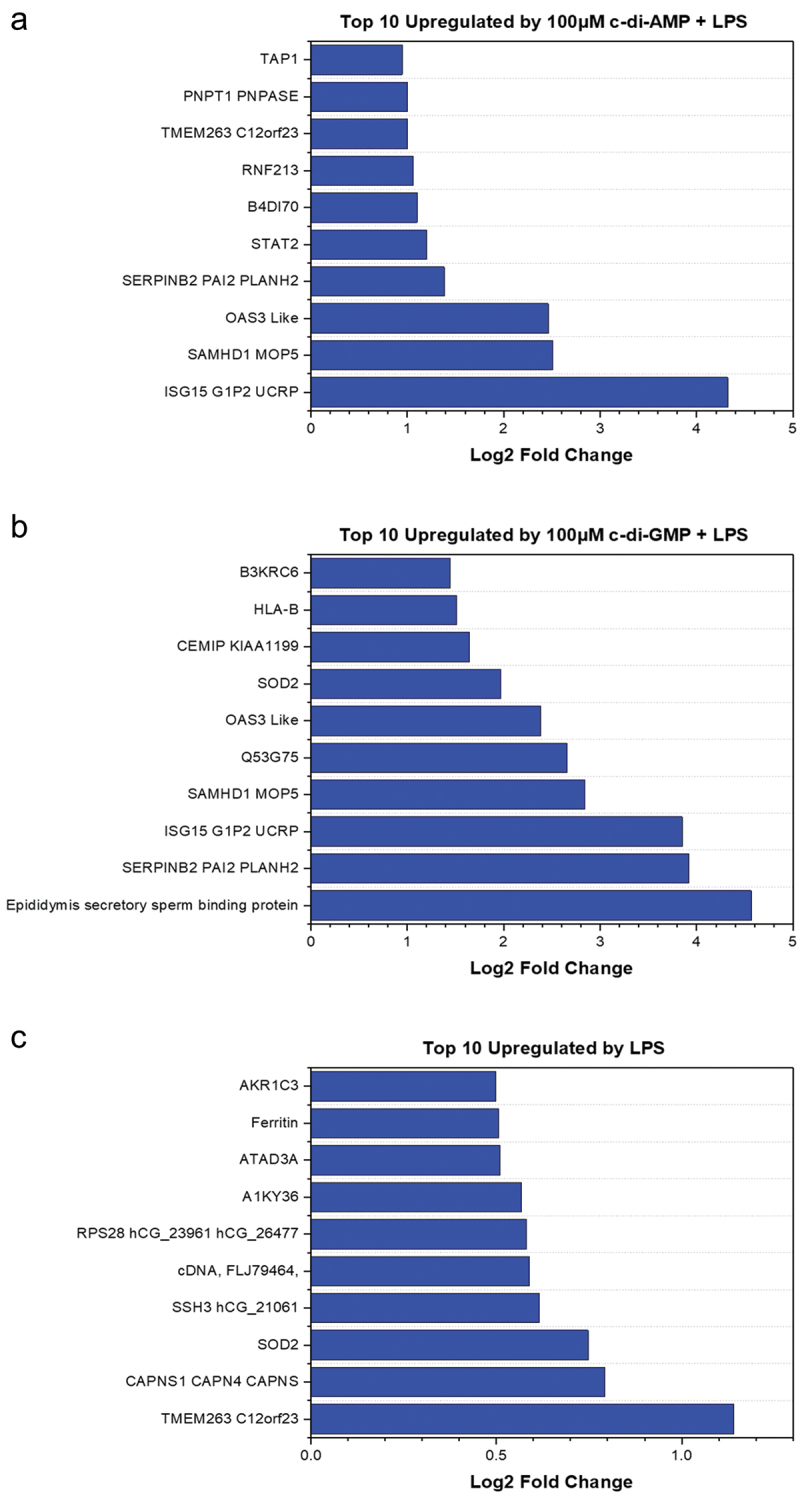
Top 10 significantly upregulated proteins with a measurable fold change in different treatment groups (a-c). Charts were plotted using the origin (pro), version 2020 software (OriginLab corporation, Northampton, MA).

The innate immunity-related proteins were amongst the 41 proteins (Table S1, Figure 2) commonly upregulated in either c-di-AMP+*Pg* LPS or c-di-GMP+*Pg* LPS treatments. Most highly upregulated proteins were ubiquitin-like protein ISG15 (ISG15), deoxynucleoside triphosphate triphosphohydrolase (SAMHD1), 2’−5’-oligoadenylate synthetase 3 (OAS3), signal transducer and activator of transcription 1-alpha/beta (STAT1), MHC class I antigen (HLA-A), interferon gamma-inducible protein 16, interferon-induced GTP-binding protein Mx (MX)1, MX2, interferon-induced protein with tetratricopeptide repeat (IFIT)1, and IFIT3.

Nevertheless, c-di-AMP+*Pg* LPS exclusively upregulated proteins that act as an anti-inflammatory agent or essential to the host cellular defense. For instance, 2′,3′-cyclic nucleotide 3′-phosphodiesterase, E3 ubiquitin-protein ligase RNF213, and D-dopachrome tautomerase were upregulated by only c-di-AMP+*Pg* LPS treatment. Interestingly, c-di-AMP+*Pg* LPS also upregulated other proteins which are not primary inflammatory proteins such as guanine nucleotide-binding protein G(I)/G(S)/G(O) subunit gamma-12 (GNG12), programmed cell death protein 8, protein phosphatase 1, adenylate kinase 2, and DNA repair protein XRCC1. On the other hand, proteins essential for immune and inflammatory responses were upregulated by c-di-GMP+*Pg* LPS, including NF-κB p100 subunit, interferon-induced guanylate-binding protein 1, MAPK protein, and TNF receptor superfamily member 11B (TNFRSF11B). Thrombospondin 1 and 2, which regulate antitumor immunity and stimulate migration of tumor cells, were also upregulated exclusively by c-di-GMP+*Pg* LPS. NF-κB essential modulator and ATP-dependent RNA helicase DDX1, which regulate host innate immune and inflammatory responses were upregulated by *Pg* LPS alone (without CDNs) [37]. These two proteins were no longer upregulated in the presence of either c-di-AMP or c-di-GMP with *Pg* LPS. The top 10 significantly upregulated proteins in each treatment group are illustrated in Figure 2a-c. For example, ISG15, OAS3 like, SAMHD1, and plasminogen activator inhibitor 2 (SERPINB2) were upregulated by both c-di-AMP+*Pg* LPS and c-di-GMP+*Pg* LPS treatments. ISG15 was the most upregulated protein by c-di-AMP+*Pg* LPS (Log2 fold change > 4). A list of significantly upregulated proteins with a measurable fold change in their different treatment groups is provided separately in Table S2.

**Figure 3.**
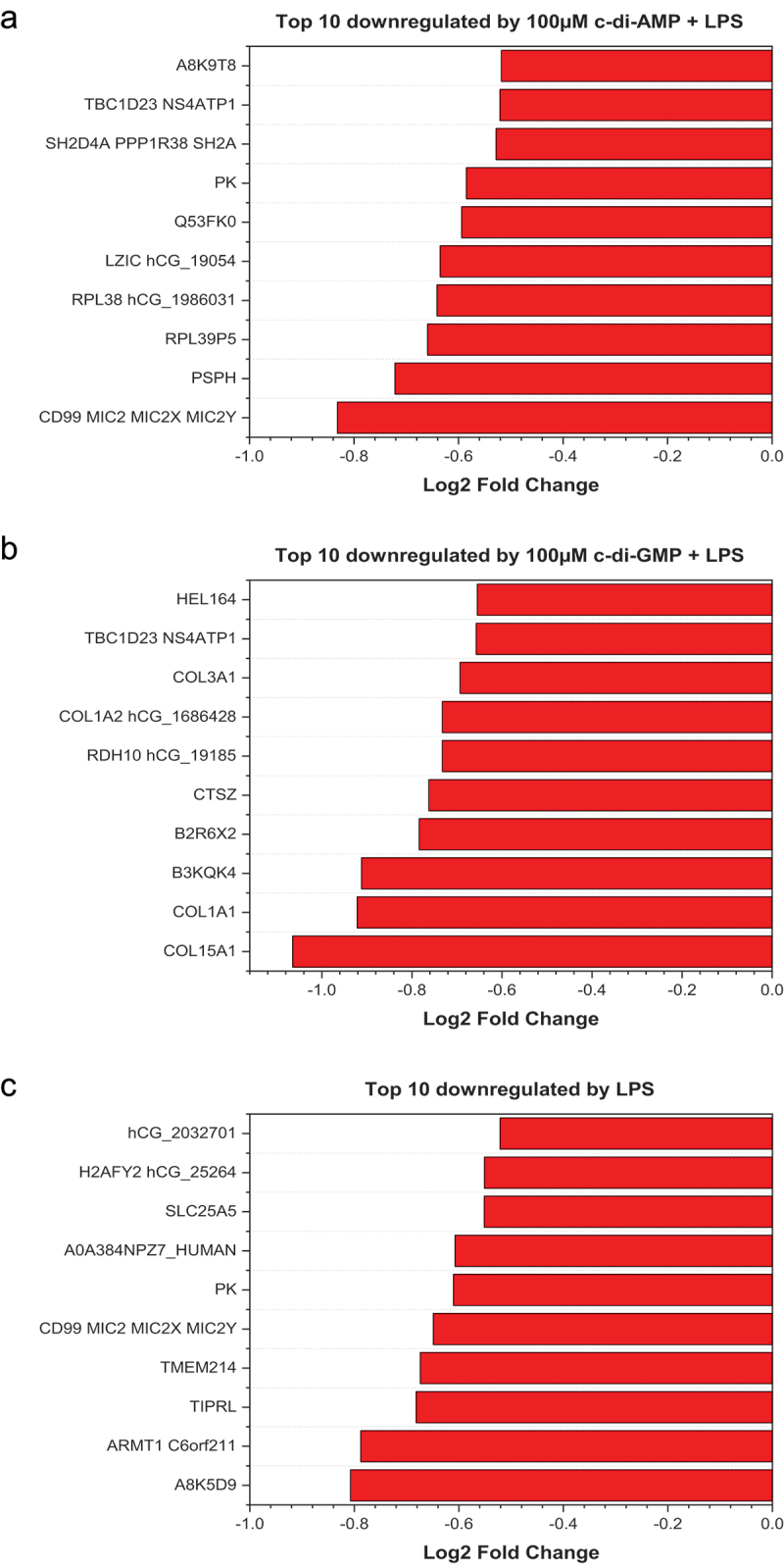
Top 10 significantly downregulated proteins with a measurable fold change in different treatment groups (A-C). Charts were plotted using the origin (pro), version 2020 software (OriginLab corporation, Northampton, MA).

The top 10 downregulated proteins are shown in Figure 3a-c. Proteins were identified as downregulated based on a measurable fold change (*p* ≤ 0.05 and Log2 fold change ≥ −0.5) and proteins that were not found in each treatment group but identified in control. Serine/threonine-protein phosphatase 2A 56 kDa regulatory subunit, phosphoserine phosphatase, and Ras-related protein R-Ras2 were downregulated by only c-di-AMP+*Pg* LPS. Glycerol-3-phosphate phosphatase (G3PP) was downregulated by only c-di-GMP+*Pg* LPS, whereas guanine nucleotide-binding protein G(i) subunit alpha (G(i) alpha-3 that regulates various pathologic processes [38], and STING were downregulated in HGFs treated by *Pg* LPS only. However, the nuclear pore complex protein Nup88, proliferating cell nuclear antigen, and IQ motif containing GTPase activating protein 3 were downregulated in HGFs treated with either c-di-AMP+*Pg* LPS or c-di-GMP+*Pg* LPS. All downregulated proteins in the three different treatment groups are provided in Table S3. A8K5D9, highly similar to Homo sapiens anillin was the most measurable downregulated protein in the *Pg* LPS only group. All downregulated proteins with a measurable fold change (*p* ≤ 0.05 and Log2 fold change ≥ −0.5) are listed with their fold change in Table S4.

**Figure 4.**
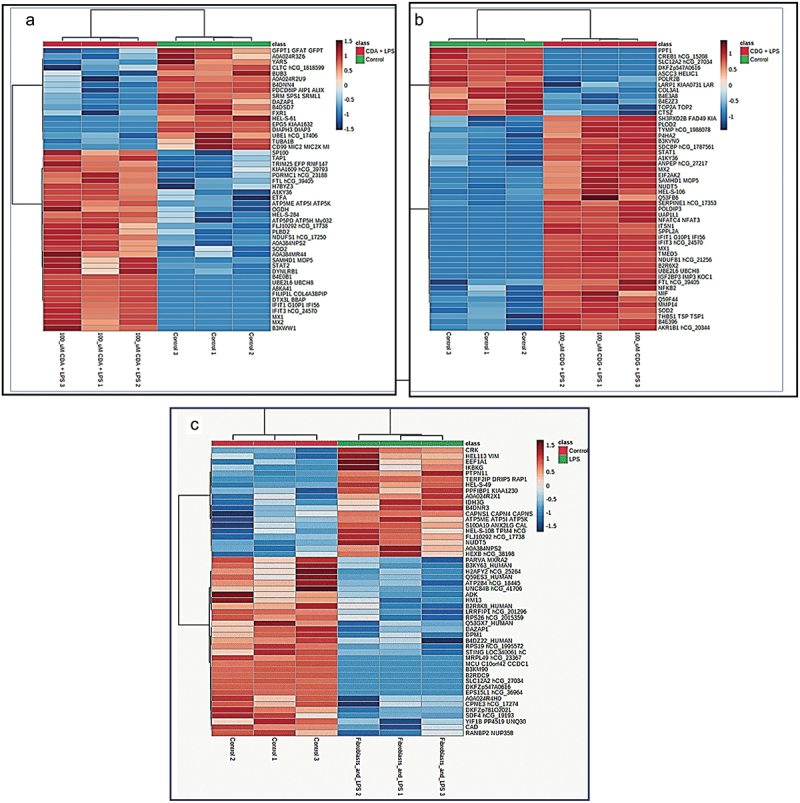
Heatmap showing the top 50 expressed proteins in three groups (blue = downregulated, oxblood/red = upregulated) after treatment of fibroblasts by (a) c-di-AMP+*Pg* LPS, (b) c-di-GMP+*Pg* LPS, (c) *pg* LPS. MetaboAnalyst software version 5.0 with auto-scale normalized data was used to plot the heatmaps. CDA+LPS = c-di-AMP+*Pg* LPS; CDG+LPS = c-di-GMP+*Pg* LPS; LPS = *pg* LPS.

Heatmaps were used to demonstrate the top 50 differentially expressed proteins in the three treatment groups (Figure 4a-c).

### Multivariate analysis of proteins identified in the three treatment conditions

Significant differentially expressed proteins (Log2 fold change ≥ ±0.5 and *p* ≤ 0.05) or exclusively identified in either control or treated HGFs were included in the multivariate analysis. The difference in the effect of c-di-AMP+*Pg* LPS, c-di-GMP+*Pg* LPS and *Pg* LPS compared to control are presented in Figure S1.

**Figure 5.**
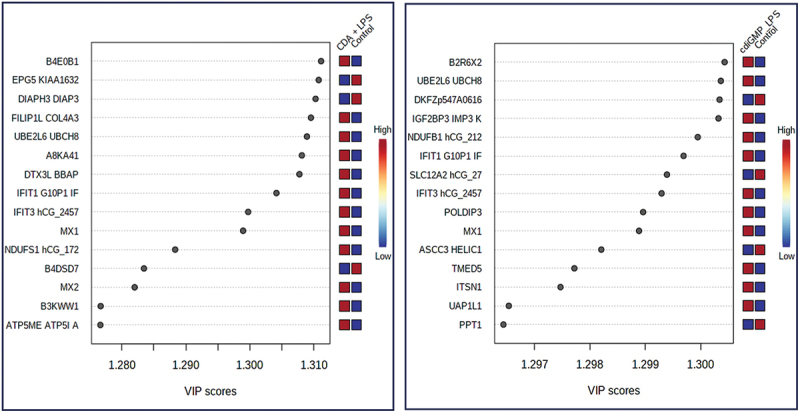
Variable importance plot (VIP) identified by PLS-DA. The colored boxes on the right (red = upregulated, blue = downregulated) indicate the corresponding concentrations of the proteins in each group. CDA+LPS = c-di-AMP+*Pg* LPS; CDG+LPS = c-di-GMP+*Pg* LPS; LPS =*Pg* LPS.

Variable importance plot (VIP) score analysis showed that c-di-AMP+*Pg* LPS as well as c-di-GMP+*Pg* LPS treatments caused an upregulation of proteins linked to inflammation, such as ubiquitin conjugating enzyme E2L6 (UBE2L6), IFIT1, IFIT3, MX1, and M×2 (Figure 5). These proteins were also at the top of statistically significant proteins (*p* < 0.0001) upregulated in each of c-di-AMP+*Pg* LPS or c-di-GMP+*Pg* LPS treated HGFs (Figures S2 and S3). Same proteins were likewise prominent among the top 50 proteins upregulated by c-di-AMP+*Pg* LPS or c-di-GMP+*Pg* LPS presented by heatmaps (Figure 4a,b).

### Effects of cyclic dinucleotides combined with LPS on signaling pathways

**Figure 6.**
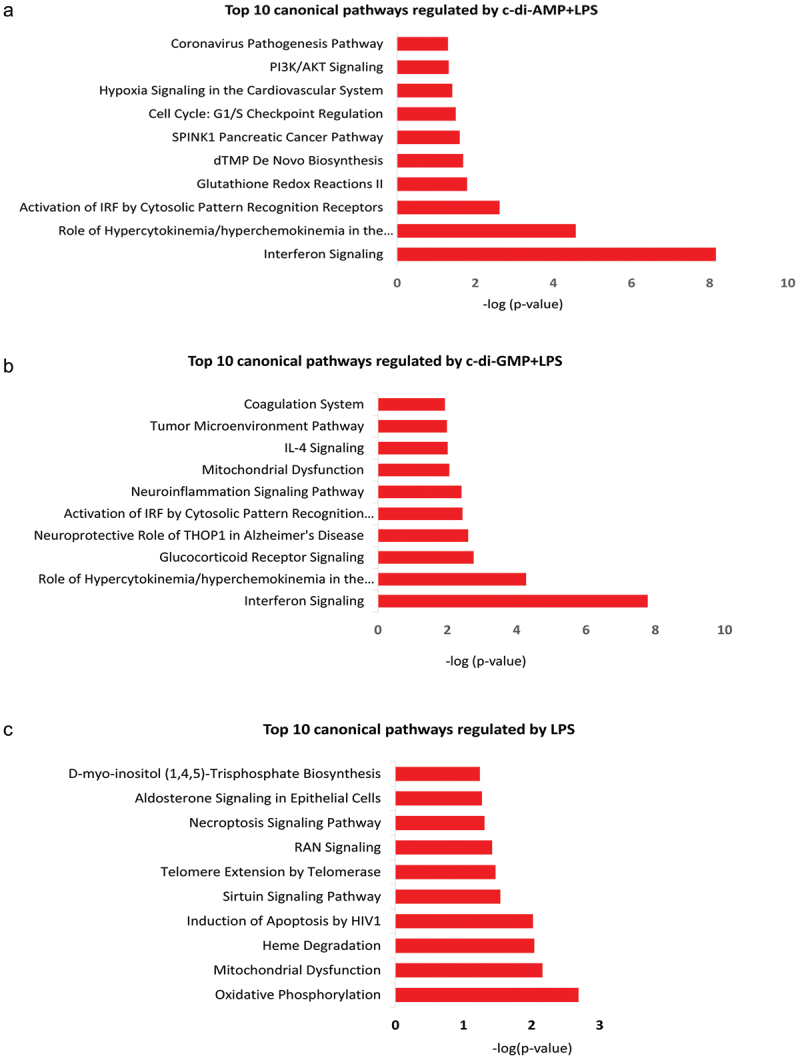
Top 10 ingenuity canonical pathways significantly regulated by (a) c-di-AMP+*Pg* LPS, (b) c-di-GMP+*Pg* LPS, (c) *pg* LPS.

Ingenuity pathway analysis (IPA) functional network core analysis revealed various pathways of expressed proteins in HGFs treated with CDNs and *Pg* LPS. C-di-AMP+*Pg* LPS significantly regulated 10 pathways with -log p > 1.3 (p < 0.05). Meanwhile, c-di-GMP+*Pg* LPS and *Pg* LPS alone significantly regulated 30 and eight pathways, respectively. C-di-AMP+*Pg* LPS treatment regulated five pathways not shown to be significantly regulated by other treatment groups, including SPINK1 pancreatic cancer pathway and cell cycle: G1/S checkpoint regulation pathway (Table S5). Notably, c-di-GMP+*Pg* LPS regulated 23 pathways not regulated by *Pg* LPS alone or in the presence of c-di-AMP, such as glucocorticoid receptor signaling, superoxide radicals degradation and hepatic fibrosis signaling (see a full list in Table S6). Likewise, *Pg* LPS alone regulated six pathways which were not significantly regulated in the presence of any of the CDNs (see Table S7). Five pathways were similarly regulated by both c-di-AMP+*Pg* LPS and c-di-GMP+*Pg* LPS groups (Table 1). Of these, interferon signaling was the most significantly regulated canonical pathway in the c-di-AMP+*Pg* LPS and c-di-GMP+*Pg* LPS groups (with a -log *p* value of 8.16 and 7.78, respectively), and with a z-score greater than 2 in both groups (Table 1). Based on a -log *p* value < 0.05, the top 10 most significantly regulated pathways in the three treatment groups are presented in Figures 6a-c. Molecules involved in commonly regulated pathways by CDNs+*Pg* LPS treated fibroblasts are shown in Table 1. The activation or inhibition role of proteins involved in signaling pathways is presented in Figure S4.

**Table 1.** Pathways significantly regulated by c-di-AMP+*Pg* LPS and c-di-GMP+*Pg* LPS with -log p > 1.3 (p < 0.05).

	Ingenuity Canonical Pathway	C-di-AMP+*Pg* LPS (-log p-value)	C-di-AMP+*Pg* LPS (z-score)	Molecules in C-di-AMP+*Pg* LPS treated fibroblasts	C-di-GMP+*Pg* LPS (-log p-value)	C-di-GMP+*Pg* LPS (z-score)	Molecules in C-di-GMP+*Pg* LPS treated fibroblasts
1	Interferon signaling	8.16	2.236	IFIT1, IFIT3, IRF9, ISG15, MX1, STAT2	7.78	2.236	IFIT1, IFIT3, IRF9, ISG15, MX1, STAT2
2	Role of hypercytokinemia/ hyperchemokinemia in the pathogenesis of influenza	4.57	2.236	IFIT3, IRF9, ISG15, MX1, STAT2	4.27	2.236	IFIT3, IRF9, ISG15, MX1, STAT2
3	Activation of IRF by cytosolic pattern recognition receptors	2.62	N	IRF9, ISG15, STAT2	2.44	N	IRF9, ISG15, STAT2
4	Hypoxia signaling in the cardiovascular system	1.41	N	UBE2A, UBE2L6	1.3	N	CREB1, UBE2L6
5	Coronavirus pathogenesis pathway	1.3	N	IRF9, SMAD3, STAT2	1.81	0	IRF9, NPC1, SERPINE1, STAT2

## Discussion

Here, global proteomics was used to provide an unbiased view of how the simultaneous exposure of gingival fibroblasts to *Pg* LPS and bacterial derived CDNs affects the proteome of the exposed cells. According to our results, simultaneous activation of HGFs with CDNs and *Pg* LPS significantly upregulated interferon signaling and innate immune -̵proteins, such as ISG15, SAMHD1, OAS3, STAT1, IRF-9, MX1, MX2, IFIT 1, IFIT 3, and DTX3L. To our knowledge, no previous study examined the cellular response of HGFs against the simultaneous challenge by CDNs and *Pg* LPS.

The present study was a continuation of our previous studies aimed to demonstrate the cellular response of gingival fibroblasts against bacterial CDNs [24,25]. The current study is the first to analyze the effects of simultaneous *Pg* LPS and bacterial derived CDNs exposures on gingival fibroblasts’ proteome response. With this, we were able to observe the possible synergistic, additive, or antagonistic activities of multiple MAMPs, especially since both LPS and cyclic dinucleotides molecular signaling can feed into the expression of key interferon products. Our previous work [24] focused only on the stimulatory effects of cyclic dinucleotides, which operate via STING-receptor to modulate the expressions of cytokines, interferons, and other immune-related proteins. While the host cells encounter diverse MAMPs, especially during infections, most studies have studied the effect of single MAMPs on host cells, including our earlier report [24]. Indeed, MAMPs may demonstrate synergistic or antagonistic effects on each other’s immune-regulatory functions. As demonstrated in another study, simultaneous signaling through TLR4 and STING leads to optimal innate immune responses by co-activating NF-κB and IRF3. This combined activation of both pathways may have a therapeutic potential [39]. In the current work, we stimulated host cells with CDNs and LPS simultaneously, as the first one activates the STING receptor while the latter one activates TLR-4 [40,41].

In the current study, CDNs were used at a concentration of 100 μM. Indeed, it is possible that the tested concentration may have undetected impacts on cells, especially those that can be observed in the periodontal environment. On the other hand, to our knowledge, concentrations of CDNs in oral biofilms, gingival crevicular fluid, and in gingival cells have not been described until now. For that reason, it is not possible to test a relevant CDN concentration on HGFs to mimic conditions in periodontitis. For that reason, we tested the CDN concentration that induces the highest IFN signal in human macrophages and binds bacterial receptors to regulate motility and biofilm formation without being cytotoxic [33,42–44].

Notably, it was suggested that c-di-AMP and c-di-GMP can not easily get into cells because they are negatively charged [45], and CDNs get into the cell with measurable impact at concentrations as high as 100 μM [24,25,27,31]. In line with this observation, high cyclic dinucleotide concentrations were used to activate immune cells in animal studies [46–48]. On the contrary, there is evidence that bacterial CDNs at concentrations less than 50 μM can also get through the cell membrane of human cells and initiate cellular response [44,49–53]. Confirmingly, we also proved that bacterial CDNs induce cellular responses in human gingival cells at concentrations of 1 and 10 μM [25,27].

In order to mimic chronic infection and constant exposure to bacterial MAMPs as seen in periodontal diseases, we also chose a 24 h time period for cell exposure to CDNs and *Pg* LPS.

Gene expression analysis of gingival biopsies from patients diagnosed with periodontitis revealed upregulated lymphocyte-related genes acting as markers of adaptive immunity [54]. In another study, the innate immunity markers were top-upregulated genes in HGFs treated by *Pg* LPS [55]. This is consistent with the present study where HGFs treated with *Pg* LPS and CDNs upregulated proteins that play significant roles in interferon signaling and innate immunity. The comparison between the number of proteins upregulated by CDNs, as found in our previous study [24], and those upregulated by simultaneous exposure to *Pg* LPS and CDNs in the present study revealed clear differences; when HGFs were treated together with *Pg* LPS and c-di-AMP, the number of upregulated proteins was increased from 46 to 93 proteins, whereas in the simultaneous presence of *Pg* LPS and c-di-GMP, no marked change was observed (77 and 72 upregulated proteins).

Research on the effect of these two groups of MAMPs, LPS, and bacterial CDNs, on HGFs and their crosstalk will allow the evaluation of their possible synergistic, additive, or antagonistic activity. Both LPS and CDN molecular signaling can feed into the expression of key interferon products. Cyclic GMP-AMP (cGAMP) that is produced by binding cytoplasmic double-stranded DNA to cyclic GMP‐AMP synthase (cGAS) and bacterial cyclic dinucleotides (c-di-AMP and c-di-GMP) bind and activate STING, which activates TBK1 and IκB kinase (IKK) [56,57]. Then, TBK1‐mediated phosphorylation and transcription of IRF3 and NF-κB inhibitor IκBα [34]. IKK phosphorylate the inhibitory IκB and activates the NF-κB. Phosphorylated IRF3 and NF-κB translocate to the nucleus, stimulating the expression of type I interferons and proinflammatory cytokines [56,57].

**Figure 7.**
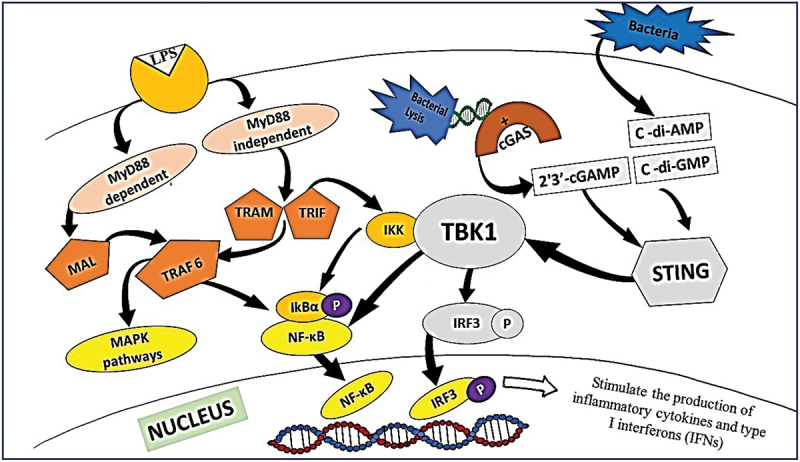
Current understanding of the signaling pathways activated by lipopolysaccharides and cyclic dinucleotides. Both STING and MyD88-independent pathways feed into expression of type I interferon through TBK1.

LPS induces innate immune responses by stimulating TLR4, and together they form a receptor complex and produce subsequent signals subdivided into early MyD88-dependent and later MyD88-independent (TRIF-dependent) [57]. MyD88-dependent and Mal adapters result in early and rapid activation of NF-κB and MAPK kinase pathways [58]. MyD88-independent by adapters TIR-domain-containing adapter-inducing interferon-β (TRIF) and TRIF-related adapter molecule (TRAM) feed into the expression of inflammatory cytokines and type I interferons [58,59]. As a later response to LPS, TLR4 activates TNF receptor-associated factor 6 (TRAF6) and TBK1. TBK1 with IKKε causes phosphorylation and nuclear translocation of IRF3; in addition, TRAF6 activation results in later activation of NF-κB by IKK α/β [58,60]. NF-κB and IRF3 induce the expression of inflammatory cytokines and type I interferons [59]. TBK1 is the central mediator of STING and MyD88-independent pathways required for transcription of type I interferons (Figure 7). While CDNs and LPS are part of different signaling complexes, they both converge on TBK1 to induce activation of IRF3 and the production of type I interferons. In addition, LPS promotes the perinuclear translocation of STING and the nuclear translocation of IRF3 [61]. This might indicate complex crosstalk between two distinct signaling pathways and explain the interaction effect observed in the present study.

C-di-AMP+*Pg* LPS upregulates GNG12, which plays a role in the inflammatory process and modulates the immune response by blocking the LPS response and activating NF-κB signaling [62,63]. An interesting finding was that c-di-AMP+*Pg* LPS upregulated the DNA repair protein XRCC1, which is not one of the primary inflammatory proteins. DNA repair protein XRCC1 is needed for the activity of DNA ligase III and its role in DNA base excision repair [64], indicating that the combined impact of CDNs and *Pg* LPS can take part in the repair of damaged DNA.

In our study, c-di-GMP+*Pg* LPS upregulated proteins related to innate and adaptive immune functions, for instance, NF-κB p100 subunit. The transcription factor NF-κB is involved in regulating innate immune cells and inflammatory T cells [65,66]. NF-κB regulates the transcription of genes related to the immune response, which consequently evokes interferons and proinflammatory cytokine production [67–69]. Other aspects with the involvement of NF-κB include, for example, apoptosis, tumorigenesis, inflammation, and various autoimmune diseases [65,66]. NF-κB2 precursor protein, p100, works similarly to an IκB-like protein with NF-κB inhibitory functions [70]. p100 is involved in the activation of noncanonical NF-κB that serves like supplementary signaling with canonical NF-κB pathway to regulate specific functions of the adaptive immune system [70,71]. In dendritic cells, p100 inhibits c-Rel (member of NF-κB) and reduces the expression of IL-23 [71].

LPS stimulates fibroblasts and inflammatory cells to produce IL-1β and TNF-α to activate osteoclast formation and bone resorption. These cytokines also increase TNFRSF11B mRNA expression [72], which is a suppressor of osteoclast activity. In the present study, TNFRSF11B mRNA protein expression was upregulated by c-di-GMP+*Pg* LPS. As observed in our recent study [24], c-di-GMP without the effect of the LPS proved to upregulate NF-κB p100 and TNFRSF11B, while in the current study, they were upregulated by c-di-GMP+*Pg* LPS but not by *Pg* LPS alone. Furthermore, c-di-GMP+*Pg* LPS enhanced the production of interferon-induced guanylate-binding protein 1. Previously, it has been shown that the induction of a class of interferon-induced GTPases (guanylate-binding proteins) has a defensive role against pathogens intracellularly [73,74] and mediates the migration of dental pulp stem cells to the inflammatory site by interferon-γ [75]. In comparison to CDNs, *Pg* LPS-treated HGFs upregulated NF-κB essential modulator, the main part of the IκB kinase complex, that controls the involvement of NF-κB signaling in the activation of many processes like inflammation, immunity, and cell survival [66]. The current study presented significantly upregulated common innate immunity-related proteins ISG15, STAT1, and HLA-A in HGFs. These proteins have a significant impact on immunity; for example, ISG15 can regulate immune modulation and upregulation of the type I interferons pathway [76], and a low level of ISG15 May indicate an increased susceptibility to periodontal inflammation [77]. IFNβ may enhance the secretion of ISG15 and its downstream cytokine, IL-10, in LPS-stimulated macrophages. In gingival tissues from patients with periodontitis, an increased proportion of macrophages and elevated expression levels of IFNβ, ISG15, and IL-10 were observed [78]. In addition, gene set enrichment analysis suggested that periodontal infection is associated with the upregulation of IFNβ, ISG15, and IL-10 [78]. While HLA-A is involved in the presentation of antigens to be recognized by cytotoxic CD8+ T cells [79,80]. Chowdhury et al. (2017) and Firatli et al. (1996) demonstrated that HLA-A functions as a protective factor against chronic periodontitis by being associated with disease resistance [81,82]. STAT mediates interferon signaling and has a key role in the expression of genes related to antibacterial function, cell survival, and pathogen response [83,84]. In patients with periodontitis, reduced *STAT1* gene expression impairs downstream IFN-I signaling, leading to diminished IFN-I activation and excessive periodontal inflammation [85]. Another study showed that *P. gingivalis* disrupts the pro-inflammatory signaling pathway by inactivating STAT1 and IRF in epithelial cells, neutrophils, and monocytes. This disruption leads to T cell imbalance and elevates the production of pro-inflammatory cytokines IL-6 and IL-23, thereby intensifying the inflammatory response and contributing to bone loss [86].

In the present study, other interferon signaling proteins, such as IFIT3, IRF-9, DTX3L, Mx1, Mx2, and IFIT1, were upregulated with a measurable fold change by both c-di-AMP+*Pg* LPS and c-di-GMP+*Pg* LPS stimulations. It is known that the gene expressions of *MX1* and *IFIT1* in peripheral blood neutrophils of individuals with periodontitis are upregulated [87]. Moreover, DTX3L expressions in peripheral blood mononuclear cells are increased in response to *P. gingivalis* infection [88]. We previously showed that IFIT3, IRF-9, DTX3L, Mx1, Mx2, and IFIT1 were upregulated in HGFs treated by CDNs in the absence of *Pg* LPS [24]. Thus, it appears that IFIT3, IRF-9, DTX3L, Mx1, Mx2, and IFIT1 upregulation in fibroblast responses are mainly regulated by CDNs than *Pg* LPS.

The combination of CDNs with *Pg* LPS significantly regulated the interferon pathway, an effect not observed in HGFs exposed to *Pg* LPS alone. IFNs trigger a wide range of biological responses, such as antiviral responses, immune surveillance, inflammation, and apoptosis [89]. Interferon signal has a critical function in antibacterial host responses by activating transcription of Janus kinases - STAT signaling, and expression of interferon-stimulated genes [90]. Type I IFN, in particular, plays a key role in regulating inflammation and is associated with various inflammatory diseases, such as systemic lupus erythematosus, rheumatoid arthritis, and periodontitis. In periodontitis patients, a reduced expression of the STAT1 gene has been observed, which diminishes IFN-I activation and leads to increased periodontal inflammation [85]. In the present study, c-di-AMP+*Pg* LPS and c-di-GMP+*Pg* LPS stimulated the interferon signal and ISG15 protein expression, suggesting an indirect effect of CDNs to inhibit bacterial survival.

C-di-GMP+*Pg* LPS significantly regulated glucocorticoid receptor signaling which regulates genes controlling the immune response. Glucocorticoid receptor has a role in inhibiting inflammatory diseases by mediating the transcription of anti-inflammatory genes [91]. Due to its anti-inflammatory, anti-proliferative, pro-apoptotic, and anti-angiogenic effects, glucocorticoid signaling plays a significant role in enhancing therapeutic strategies for various diseases and contributing to developing more effective therapeutic strategies [92].

C-di-AMP+*Pg* LPS upregulated cell cycle: G1/S checkpoint regulation pathway, which acts as a DNA surveillance mechanisms to prevent the accumulation and propagation of genetic errors during cell division. By checkpoints or in response to irreparable DNA damage, cell cycle progression can be delayed or induce cell cycle exit or cell death [93]. Permanent genomic alterations can occur due to the propagation of DNA lesions caused by the loss of checkpoint integrity [94].

Moreover, our study shows that c-di-AMP+*Pg* LPS also significantly regulated non-inflammatory pathways such as SPINK1 pancreatic cancer pathway. SPINK1, a protease inhibitor, is essential in many physiological events, such as tissue differentiation, apoptosis modulation, and tissue maintenance and repair. In addition to its contribution in reproduction, it contributes to pathological processes and can be defined as a diagnostic marker due to its elevation in many cancers [95,96].

To conclude, the combined effects of MAMPs (CDNs and *Pg* LPS) on human gingival fibroblasts lead to the activation of the interferon signaling pathway and related immune proteins such as ISG15, STAT1, and HLA-A. This interaction highlights the significant impact of MAMPs on stimulating antimicrobial cellular responses in these cells.

## Supplementary Material

Table_S4_Down_regulated_proteins.pdf

Table_S3_Down_regulated_proteins.pdf

Table_S1_up_regulated_proteins.pdf

Table_S2_up_regulated_proteins.pdf

Supplementary material.pdf

## References

[1] Phipps RP, Borrello MA, Blieden TM. Fibroblast heterogeneity in the periodontium and other tissues. J Periodontal Res. 1997;32(1):159–15. doi: 10.1111/j.1600-0765.1997.tb01398.x 9085227

[2] Naylor AJ, Filer A, Buckley CD. The role of stromal cells in the persistence of chronic inflammation. Clin Exp Immunol. 2013;171(1):30–35. doi: 10.1111/j.1365-2249.2012.04634.x 23199320 PMC3530092

[3] Morandini AC, Sipert CR, Ramos-Junior ES, et al. Periodontal ligament and gingival fibroblasts participate in the production of tgf-beta, interleukin (IL)-8 and IL-10. Braz Oral Res. 2011;25(2):157–162. doi: 10.1590/S1806-83242011000200010 21537641

[4] Jang JH, Shin HW, Lee JM, et al. An overview of pathogen recognition receptors for innate immunity in dental pulp. Mediators Inflamm. 2015;2015(1):794143. doi: 10.1155/2015/794143 26576076 PMC4630409

[5] Zhong Y, Kinio A, Saleh M. Functions of NOD-Like receptors in human diseases. Front Immunol. 2013;4:333. doi: 10.3389/fimmu.2013.00333 24137163 PMC3797414

[6] Medzhitov R. Recognition of microorganisms and activation of the immune response. Nature. 2007;449(7164):819–826. doi: 10.1038/nature06246 17943118

[7] Subramani T, Rathnavelu V, Alitheen NB, et al. Cellular crosstalk mechanism of toll-like receptors in gingival overgrowth (review). Int J Mol Med. 2015;35(5):1151–1158. doi: 10.3892/ijmm.2015.2144 25812632

[8] Bautista-Hernández LA, Gómez-Olivares JL, Buentello-Volante B, et al. Fibroblasts: the unknown sentinels eliciting immune responses against microorganisms. Eur J Microbiol Immunol (Bp). 2017;7(3):151–157. doi: 10.1556/1886.2017.00009 29034104 PMC5632742

[9] Mogensen TH. Pathogen recognition and inflammatory signaling in innate immune defenses. Clin Microbiol Rev. 2009;22(2):240–273. doi: 10.1128/CMR.00046-08 19366914 PMC2668232

[10] Liu J, Wang Y, Ouyang X. Beyond toll-like receptors: *porphyromonas gingivalis* induces IL-6, IL-8, and VCAM-1 expression through nod-mediated nf-κB and ERK signaling pathways in periodontal fibroblasts. Inflammation. 2014;37(2):522–533. doi: 10.1007/s10753-013-9766-0 24162780

[11] Bozkurt SB, Hakki SS, Hakki EE, et al. *Porphyromonas gingivalis* lipopolysaccharide induces a proinflammatory human gingival fibroblast phenotype. Inflammation. 2017;40(1):144–153. doi: 10.1007/s10753-016-0463-7 27812843

[12] Olsen I, Singhrao SK. Importance of heterogeneity in *Porhyromonas gingivalis* lipopolysaccharide lipid a in tissue specific inflammatory signalling. J Oral Microbiol. 2018;10(1):1440128. doi: 10.1080/20002297.2018.1440128 29503705 PMC5827638

[13] Wang PL, Ohura K. *Porphyromonas gingivalis* lipopolysaccharide signaling in gingival fibroblasts-CD14 and Toll-like receptors. Crit Rev Oral Biol Med. 2002;13(2):132–142. doi: 10.1177/154411130201300204 12097356

[14] Yan H, Chen W. The promise and challenges of cyclic dinucleotides as molecular adjuvants for vaccine development. Vaccines (Basel). 2021;9(8):917. doi: 10.3390/vaccines9080917 34452042 PMC8402453

[15] Mahla RS, Reddy MC, Prasad DV, et al. Sweeten PAMPs: role of sugar complexed PAMPs in innate immunity and vaccine biology. Front Immunol. 2013;4:248. doi: 10.3389/fimmu.2013.00248 24032031 PMC3759294

[16] Weber F. Antiviral innate immunity: introduction. Reference Module In Biomed Sci. 2014. doi:10.1016/B978-0-12-801238-3.02608-8.

[17] da Aline Dias P, de Athalia Marins A, Gabriel Guarany de A, et al. The world of cyclic Dinucleotides in bacterial behavior. Molecules. 2020;25(10):2462. doi: 10.3390/molecules25102462 32466317 PMC7288161

[18] Danilchanka O, Mekalanos JJ. Cyclic dinucleotides and the innate immune response. Cell. 2013;154(5):962–970. doi: 10.1016/j.cell.2013.08.014 23993090 PMC3931520

[19] Römling U, Galperin MY, Gomelsky M. Cyclic di-gmp: the first 25 years of a universal bacterial second messenger. Microbiol Mol Biol Rev. 2013;77(1):1–52. doi: 10.1128/MMBR.00043-12 23471616 PMC3591986

[20] Gürsoy UK, Gürsoy M, Könönen E, et al. Cyclic dinucleotides in oral bacteria and in oral Biofilms. Front Cell Infect Microbiol. 2017;7:273. doi: 10.3389/fcimb.2017.00273 28680857 PMC5478684

[21] Chaudhuri S, Pratap S, Paromov V, et al. Identification of a diguanylate cyclase and its role in *Porphyromonas gingivalis* virulence. Infect Immun. 2014;82(7):2728–2735. doi: 10.1128/IAI.00084-14 24733094 PMC4097614

[22] Bian J, Liu X, Cheng YQ, et al. Inactivation of cyclic Di-gmp binding protein TDE0214 affects the motility, biofilm formation, and virulence of *treponema denticola*. J Bacteriol. 2013;195(17):3897–3905. doi: 10.1128/JB.00610-13 23794624 PMC3754597

[23] Decout A, Katz JD, Venkatraman S, et al. The cGAS-sting pathway as a therapeutic target in inflammatory diseases. Nat Rev Immunol. 2021;21(9):548–569. doi: 10.1038/s41577-021-00524-z 33833439 PMC8029610

[24] Onyedibe KI, Elmanfi S, Aryal UK, et al. Global proteomics of fibroblast cells treated with bacterial cyclic dinucleotides, c-di-gmp and c-di-amp. J Oral Microbiol. 2021;14(1):2003617. doi: 10.1080/20002297.2021.2003617 34992733 PMC8725719

[25] Elmanfi S, Sintim HO, Zhou J, et al. Activation of gingival fibroblasts by bacterial cyclic dinucleotides and Lipopolysaccharide. Pathogens. 2020;9(10):792. doi: 10.3390/pathogens9100792 32993127 PMC7600373

[26] Onyedibe KI, Mohallem R, Wang M, et al. Proteomic and phosphoproteomic analyses of Jurkat T-cell treated with 2‘3’ cGAMP reveals various signaling axes impacted by cyclic dinucleotides. J Proteomics. 2023;279:104869. doi: 10.1016/j.jprot.2023.104869 36889538

[27] Elmanfi S, Zhou J, Sintim HO, et al. Regulation of gingival epithelial cytokine response by bacterial cyclic dinucleotides. J Oral Microbiol. 2018;11(1):1538927. doi: 10.1080/20002297.2018.1538927 30598733 PMC6263105

[28] Oksanen J, Hormia M. An organotypic in vitro model that mimics the dento-epithelial junction. J Periodontol. 2002;73(1):86–93. doi: 10.1902/jop.2002.73.1.86 11846204

[29] Gaffney BL, Veliath E, Zhao J, et al. One-flask syntheses of c-di-gmp and the [rp,rp] and [rp,sp] thiophosphate analogues. Org Lett. 2010;12(14):3269–3271. doi: 10.1021/ol101236b 20572672 PMC2905038

[30] Gaffney BL, Stebbins ND, Jones RA. Synthesis of biotinylated c-di-gmp and c-di-amp using click conjugation. Nucleosides, Nucleotides And Nucleic Acids. 2013;32(1):1–16. doi: 10.1080/15257770.2012.748196 PMC3561633 23360291

[31] Aryal UK, Hedrick V, Onyedibe KI, et al. Global proteomic analyses of STING-Positive and -negative macrophages reveal STING and Non-sting differentially regulated cellular and molecular pathways. Proteomics Clin Appl. 2020;14(3):e1900109. doi: 10.1002/prca.201900109 32065729

[32] Kim SQ, Mohallem R, Franco J, et al. Global landscape of protein complexes in postprandial-state livers from diet-induced obese and lean mice. Biochem Biophys Res Commun. 2022;629:40–46. doi: 10.1016/j.bbrc.2022.08.070 36099783

[33] Sooreshjani MA, Gursoy UK, Aryal UK, et al. Proteomic analysis of RAW macrophages treated with cGAMP or c-di-gmp reveals differentially activated cellular pathways. RSC Adv. 2018;8(64):36840–36851. doi: 10.1039/C8RA04603D 35558957 PMC9089301

[34] Opoku-Temeng C, Onyedibe KI, Aryal UK, et al. Proteomic analysis of bacterial response to a 4-hydroxybenzylidene indolinone compound, which re-sensitizes bacteria to traditional antibiotics. J Proteomics. 2019;30;202 103368. doi: 10.1016/j.jprot.2019.04.018 31028946

[35] Cox J, Mann M. MaxQuant enables high peptide identification rates, individualized p.p.b.-range mass accuracies and proteome-wide protein quantification. Nat Biotechnol. 2008;26(12):1367–1372. doi: 10.1038/nbt.1511 19029910

[36] Tyanova S, Temu T, Sinitcyn P, et al. The perseus computational platform for comprehensive analysis of (prote)omics data. Nat Methods. 2016;13(9):731–740. doi: 10.1038/nmeth.3901 27348712

[37] Ishaq M, Ma L, Wu X, et al. The dead-box RNA helicase DDX1 interacts with RelA and enhances nuclear factor kappaB-mediated transcription. J Cell Biochem. 2009;106(2):296–305. doi: 10.1002/jcb.22004 19058135

[38] Li X, Wang D, Chen Z, et al. Gαi1 and Gαi3 regulate macrophage polarization by forming a complex containing CD14 and Gab1. Proc Natl Acad Sci U S A. 2015;112(15):4731–4736. doi: 10.1073/pnas.1503779112 25825741 PMC4403188

[39] Higgs EF, Gajewski TF. Synergistic innate immune activation and anti-tumor immunity through combined STING and TLR4 stimulation. Prepr bioRxiv. 2024;2024.04.08.588610.

[40] Kabelitz D, Zarobkiewicz M, Heib M, et al. Signal strength of STING activation determines cytokine plasticity and cell death in human monocytes. Sci Rep. 2022;12(1):17827. doi: 10.1038/s41598-022-20519-7 36280676 PMC9590392

[41] Ciesielska A, Matyjek M, Kwiatkowska K. TLR4 and CD14 trafficking and its influence on lps-induced pro-inflammatory signaling. Cell Mol Life Sci. 2021;78(4):1233–1261. doi: 10.1007/s00018-020-03656-y 33057840 PMC7904555

[42] Khan F, Jeong GJ, Tabassum N, et al. Functional diversity of c-di-gmp receptors in prokaryotic and eukaryotic systems. Cell Commun Signal. 2023;21(1):259. doi: 10.1186/s12964-023-01263-5 37749602 PMC10519070

[43] Karaolis DK, Rashid MH, Chythanya R, et al. C-di-gmp (3’-5’-cyclic diguanylic acid) inhibits Staphylococcus aureus cell-cell interactions and biofilm formation. Antimicrob Agents Chemother. 2005;49(3):1029–1038. doi: 10.1128/AAC.49.3.1029-1038.2005 15728899 PMC549248

[44] Karaolis DK, Cheng K, Lipsky M, et al. 3‘,5’-cyclic diguanylic acid (c-di-gmp) inhibits basal and growth factor-stimulated human colon cancer cell proliferation. Biochem Biophys Res Commun. 2005;329(1):40–45. doi: 10.1016/j.bbrc.2005.01.093 15721270

[45] Gogoi H, Mansouri S, Jin L. The age of cyclic dinucleotide vaccine adjuvants. Vaccines (Basel). 2020;8(3):453. doi: 10.3390/vaccines8030453 32823563 PMC7563944

[46] Chandra D, Quispe-Tintaya W, Jahangir A, et al. STING ligand c-di-gmp improves cancer vaccination against metastatic breast cancer. Cancer Immunol Res. 2014;2(9):901–910. doi: 10.1158/2326-6066.CIR-13-0123 24913717 PMC4264585

[47] Karaolis DK, Newstead MW, Zeng X, et al. Cyclic di-gmp stimulates protective innate immunity in bacterial pneumonia. Infect Immun. 2007;75(10):4942–4950. doi: 10.1128/IAI.01762-06 17646358 PMC2044534

[48] Karaolis DK, Means TK, Yang D, et al. Bacterial c-di-gmp is an immunostimulatory molecule. J Immunol. 2007;178(4):2171–2181. doi: 10.4049/jimmunol.178.4.2171 17277122

[49] Steinberger O, Lapidot Z, Ben-Ishai Z, et al. Elevated expression of the CD4 receptor and cell cycle arrest are induced in Jurkat cells by treatment with the novel cyclic dinucleotide 3‘,5’-cyclic diguanylic acid. FEBS Lett. 1999;444(1):125–129. doi: 10.1016/S0014-5793(99)00036-8 10037160

[50] Luteijn RD, Zaver SA, Gowen BG, et al. SLC19A1 transports immunoreactive cyclic dinucleotides. Nature. 2019;573(7774):434–438. doi: 10.1038/s41586-019-1553-0 31511694 PMC6785039

[51] Lahey LJ, Mardjuki RE, Wen X, et al. LRRC8A: C/E heteromeric channels are ubiquitous transporters of cGAMP. Mol Cell. 2020;80(4):578–591. doi: 10.1016/j.molcel.2020.10.021 33171122 PMC12629894

[52] Ritchie C, Cordova AF, Hess GT, et al. SLC19A1 is an importer of the immunotransmitter cGAMP. Mol Cell. 2019;75(2):372–381.e5. doi: 10.1016/j.molcel.2019.05.006 31126740 PMC6711396

[53] Cordova AF, Ritchie C, Böhnert V, et al. Human SLC46A2 is the dominant cGAMP importer in extracellular cGAMP-sensing macrophages and monocytes. ACS Cent Sci. 2021;7(6):1073–1088. doi: 10.1021/acscentsci.1c00440 34235268 PMC8228594

[54] Davanian H, Stranneheim H, Båge T, et al. Gene expression profiles in paired gingival biopsies from periodontitis-affected and healthy tissues revealed by massively parallel sequencing. PLOS ONE. 2012;7(9):e46440. doi: 10.1371/journal.pone.0046440 23029519 PMC3460903

[55] Xie Y, Sun M, Xia Y, et al. An rna-seq screen of *P. gingivalis* LPS treated human gingival fibroblasts. Arch Oral Biol. 2018;88:77–84. doi: 10.1016/j.archoralbio.2018.01.002 29407755

[56] Motwani M, Pesiridis S, Fitzgerald KA. DNA sensing by the cGAS–sting pathway in health and disease. Nat Rev Genet. 2019;20(11):657–674. doi: 10.1038/s41576-019-0151-1 31358977

[57] Ablasser A, Gulen MF. The role of cGAS in innate immunity and beyond. J Mol Med. 2016;94(10):1085–1093. doi: 10.1007/s00109-016-1423-2 27154323

[58] Pålsson-McDermott EM, O’Neill LA. Signal transduction by the lipopolysaccharide receptor, toll-like receptor-4. Immunology. 2004;113(2):153–162. doi: 10.1111/j.1365-2567.2004.01976.x 15379975 PMC1782563

[59] Zhou R, Zhang Q, Xu P. TBK1, a central kinase in innate immune sensing of nucleic acids and beyond. Acta Biochim Biophys Sin (Shanghai). 2020;52(7):757–767. doi: 10.1093/abbs/gmaa051 32458982

[60] Fitzgerald KA, McWhirter SM, Faia KL, et al. Ikkepsilon and TBK1 are essential components of the IRF3 signaling pathway. Nat Immunol. 2003;4(5):491–496. doi: 10.1038/ni921 12692549

[61] Li N, Zhou H, Wu H, et al. STING-IRF3 contributes to lipopolysaccharide-induced cardiac dysfunction, inflammation, apoptosis and pyroptosis by activating NLRP3. Redox Biol. 2019;24:101215. doi: 10.1016/j.redox.2019.101215 31121492 PMC6529775

[62] Larson KC, Lipko M, Dabrowski M, et al. Gng12 is a novel negative regulator of lps-induced inflammation in the microglial cell line BV-2. Inflamm Res. 2010;59(1):15–22. doi: 10.1007/s00011-009-0062-2 19568691

[63] Li J, Jin C, Zou C, et al. GNG12 regulates PD-L1 expression by activating nf-κB signaling in pancreatic ductal adenocarcinoma. FEBS Open Bio. 2020;10(2):278–287. doi: 10.1002/2211-5463.12784 PMC6996305 31898405

[64] Caldecott KW, McKeown CK, Tucker JD, et al. An interaction between the mammalian DNA repair protein XRCC1 and DNA ligase III. Mol Cell Biol. 1994;14(1):68–76. doi: 10.1128/MCB.14.1.68 8264637 PMC358357

[65] Barnabei L, Laplantine E, Mbongo W, et al. Nf-κB: At the borders of autoimmunity and inflammation. Front Immunol. 2021;12:716469. doi: 10.3389/fimmu.2021.716469 34434197 PMC8381650

[66] Liu T, Zhang L, Joo D, et al. Nf-κB signaling in inflammation. Signal Transduct Target Ther. 2017;2(1):17023. doi: 10.1038/sigtrans.2017.23 29158945 PMC5661633

[67] Smith EM, Gregg M, Hashemi F, et al. Corticotropin releasing factor (CRF) activation of nf-κB-Directed transcription in leukocytes. Cell Mol Neurobiol. 2006;26(4–6):1019–1034. doi: 10.1007/s10571-006-9040-1 PMC11520635 16633893

[68] Akira S, Uematsu S, Takeuchi O. Pathogen recognition and innate immunity. Cell. 2006;124(4):783–801. doi: 10.1016/j.cell.2006.02.015 16497588

[69] Kawai T, Akira S. The roles of TLRs, RLRs and NLRs in pathogen recognition. Int Immunol. 2009;21(4):317–337. doi: 10.1093/intimm/dxp017 19246554 PMC2721684

[70] Beinke S, Ley SC. Functions of nf-κB1 and nf-κB2 in immune cell biology. Biochem J. 2004;382(2):393–409. doi: 10.1042/BJ20040544 15214841 PMC1133795

[71] Mise-Omata S, Obata Y, Doi TS. p100, a precursor of nf-κB2, inhibits c-rel and reduces the expression of IL-23 in dendritic cells. Biochem Biophys Res Commun. 2014;453(3):332–337. doi: 10.1016/j.bbrc.2014.09.143 25305492

[72] Sakata M, Shiba H, Komatsuzawa H, et al. Expression of osteoprotegerin (osteoclastogenesis inhibitory factor) in cultures of human dental mesenchymal cells and epithelial cells. J Bone Miner Res. 1999;14(9):1486–1492. doi: 10.1359/jbmr.1999.14.9.1486 10469276

[73] Meunier E, Broz P. Interferon-induced guanylate-binding proteins promote cytosolic lipopolysaccharide detection by caspase-11. DNA Cell Biol. 2015;34(1):1–5. doi: 10.1089/dna.2014.2701 25347553

[74] Bai S, Chen T, Deng X. Guanylate-binding protein 1 promotes migration and invasion of human periodontal ligament stem cells. Stem Cells Int. 2018;2018:1–8. doi: 10.1155/2018/6082956 PMC6304207 30622567

[75] He X, Jiang W, Luo Z, et al. Ifn-γ regulates human dental pulp stem cells behavior via nf-κB and MAPK signaling. Sci Rep. 2017;7(1):40681. doi: 10.1038/srep40681 28098169 PMC5241669

[76] Jeon YJ, Yoo HM, Chung CH. ISG15 and immune diseases. Biochim Biophys Acta. 2010;1802(5):485–496. doi: 10.1016/j.bbadis.2010.02.006 20153823 PMC7127291

[77] Bostanci N, Silbereisen A, Bao K, et al. Salivary proteotypes of gingivitis tolerance and resilience. J Clin Periodontol. 2020;47(11):1304–1316. doi: 10.1111/jcpe.13358 32777086 PMC7692908

[78] Liu J, Li T, Zhang S, et al. Proteomic and single-cell analysis shed new light on the anti-inflammatory role of interferonβ in chronic periodontitis. Front Pharmacol. 2023;14:1232539. doi: 10.3389/fphar.2023.1232539 37876725 PMC10590904

[79] Wieczorek M, Abualrous ET, Sticht J, et al. Major histocompatibility complex (MHC) class I and MHC class II proteins: conformational plasticity in antigen presentation. Front Immunol. 2017;8:292. doi: 10.3389/fimmu.2017.00292 28367149 PMC5355494

[80] Zhou F. Molecular mechanisms of ifn-gamma to upregulate MHC class I antigen processing and presentation. Int Rev Immunol. 2009;28(3–4):239–260. doi: 10.1080/08830180902978120 19811323

[81] Chowdhury M, Agrawal N, Kundu D, et al. Association of human leukocyte antigens class I and Class II antigens with chronic periodontitis in East India. J Indian Soc Periodontol. 2017;21(6):494–498. doi: 10.4103/jisp.jisp_309_16 29551870 PMC5846248

[82] Firatli E, Kantarci A, Cebeci I, et al. Association between HLA antigens and early onset periodontitis. J Clin Periodontol. 1996;23(6):563–566. doi: 10.1111/j.1600-051X.1996.tb01825.x 8811476

[83] Adámková L, Soucková K, Kovarík J. Transcription protein STAT1: biology and relation to cancer. Folia Biol (Praha). 2007;53(1):1–6. 17328836 10.14712/fb2007053010001

[84] Baran-Marszak F, Feuillard J, Najjar I, et al. Differential roles of STAT1alpha and STAT1beta in fludarabine-induced cell cycle arrest and apoptosis in human B cells. Blood. 2004;104(8):2475–2483. doi: 10.1182/blood-2003-10-3508 15217838

[85] Ji L, Li T, Chen H, et al. The crucial regulatory role of type I interferon in inflammatory diseases. Cell Biosci. 2023;13(1):230. doi: 10.1186/s13578-023-01188-z 38124132 PMC10734085

[86] Abdulkareem AA, Al-Taweel FB, Al-Sharqi AJB, et al. Current concepts in the pathogenesis of periodontitis: from symbiosis to dysbiosis. J Oral Microbiol. 2023;15(1):2197779. doi: 10.1080/20002297.2023.2197779 37025387 PMC10071981

[87] Cárdenas AM, Ardila LJ, Vernal R, et al. Biomarkers of periodontitis and its differential DNA methylation and gene expression in immune cells: a systematic review. Int J Mol Sci. 2022;23(19):12042. doi: 10.3390/ijms231912042 36233348 PMC9570497

[88] Pandi K, Angabo S, Makkawi H, et al. *P. gingivalis*-induced TLR2 interactome analysis reveals association with PARP9. J Dent Res. 2024;103(3):329–338. doi: 10.1177/00220345231222181 38344758

[89] Katsoulidis E, Kaur S, Platanias LC. Deregulation of interferon signaling in malignant cells. Pharmaceuticals (Basel). 2010;3(2):406–418. doi: 10.3390/ph3020406 27713259 PMC4033917

[90] Alphonse N, Dickenson RE, Odendall C. Interferons: tug of war between bacteria and their Host. Front Cell Infect Microbiol. 2021;11:624094. soulidis E, Kaur S, Platanias LC. Deregulation of Interferon Signaling in Malignant Cells. Pharmaceuticals (Basel). 2010;3(2):406-418. doi: 10.3389/fcimb.2021.624094 33777837 PMC7988231

[91] Kadmiel M, Cidlowski JA. Glucocorticoid receptor signaling in health and disease. Trends Pharmacol Sci. 2013;34(9):518–530. doi: 10.1016/j.tips.2013.07.003 23953592 PMC3951203

[92] Desmet SJ, De Bosscher K. Glucocorticoid receptors: finding the middle ground. J Clin Invest. 2017;127(4):1136–1145. doi: 10.1172/JCI88886 28319043 PMC5373866

[93] Grzelakowska-Sztabert B. Cell cycle checkpoints – molecular background. Folia Morphol (Warsz). 2004;63(1):1–3. 15039892

[94] Matthews HK, Bertoli C, de Bruin RAM. Cell cycle control in cancer. Nat Rev Mol Cell Biol. 2022;23(1):74–88. doi: 10.1038/s41580-021-00404-3 34508254

[95] Itkonen O, Stenman UH. TATI as a biomarker. Clin Chim Acta. 2014;431:260–269. doi: 10.1016/j.cca.2014.02.014 24583226

[96] Liao C, Wang Q, An J, et al. SPINKs in tumors: potential therapeutic targets. Front Oncol. 2022;12:833741. doi: 10.3389/fonc.2022.833741 35223512 PMC8873584

