# Supplementary material for: Activation of cellular responses by cyclic dinucleotides and porphyromonas gingivalis lipopolysaccharide: a proteomic study on gingival fibroblasts
Source: J Oral Microbiol. 2024 Dec 9;17(1):2431453. doi: 10.1080/20002297.2024.2431453 (PMC11632945; doi:10.1080/20002297.2024.2431453)
Supplement: Table_S4_Down_regulated_proteins.pdf [file ZJOM_A_2431453_SM5363.pdf]

Table S4. List of downregulated proteins with a measurable fold change (Log2 fold change ≥ -0.5 and p ≤ 0.05) in different treatment groups (100 µM c-di-AMP+*Pg* LPS or c-di-GMP+*Pg* LPS or *Pg* LPS)

S/No	Treatment/Venn Diagram group
**i.**	**35 common elements in "100 µM c-di-AMP+*Pg* LPS↓↓", "100 µM c-di-GMP+*Pg* LPS↓↓" and "*Pg* LPS↓↓":**
	Protein kinase
	TBC1 domain family member 23 (HCV non-structural protein 4A-transactivated protein 1)
	cDNA FLJ77424; highly similar to Homo sapiens anillin; actin binding protein (scraps homolog; Drosophila); mRNA
	TIP41-like protein (Putative MAPK-activating protein PM10) (Type 2A-interacting protein) (TIP)
	Threonyl-tRNA synthetase variant (Fragment)
	Epididymis secretory sperm binding protein
	cDNA; FLJ93949; highly similar to Homo sapiens NIMA (never in mitosis gene a)-related kinase 7 (NEK7); mRNA
	G1 to S phase transition 1
	Alpha-parvin (Actopaxin) (CH-ILKBP) (Calponin-like integrin-linked kinase-binding protein) (Matrix-remodeling-associated protein 2)
	Carboxypeptidase D (EC 3.4.17.22) (Metallocarboxypeptidase D) (gp180)
	Serine threonine kinase 39 isoform B (Fragment)
	cDNA FLJ60304; highly similar to Rab GTPase-binding effector protein 1
	ER lumen protein-retaining receptor 1 (KDEL endoplasmic reticulum protein retention receptor 1) (KDEL receptor 1) (Putative MAPK-activating protein PM23)
	Non-specific serine/threonine protein kinase (EC 2.7.11.1)
	SGCB protein
	Guanine nucleotide-binding protein-like 3 (E2-induced gene 3 protein) (Novel nucleolar protein 47) (NNP47) (Nucleolar GTP-binding protein 3) (Nucleostemin)
	Plasminogen receptor (KT) (Plg-R(KT))
	Ribonucloprotein (Fragment)
	Tumor protein p53-inducible protein 11 (Fragment)
	Telomere length regulation protein TEL2 homolog (Protein clk-2 homolog) (hCLK2)
	E3 ubiquitin-protein ligase RNF181
	cDNA FLJ60156; highly similar to Vacuolar protein sorting protein 52
	Abl interactor 2
	DOCK5 (Fragment)
	RUN and FYVE domain-containing protein 1 (FYVE-finger protein EIP1) (La-binding protein 1) (Rab4-interacting protein) (Zinc finger FYVE domain-containing protein 12)
	TBC1 domain family member 17 (Fragment)
	Protein pelota homolog (EC 3.1.-.-)
	Zinc finger; NFX1-type containing 1; isoform CRA_b

	Transcription elongation factor A protein-like 3 (TCEA-like protein 3) (Transcription elongation factor S-II protein-like 3)
	Neuronal protein
	Transforming acidic coiled-coil-containing protein 3
	cAMP-regulated phosphoprotein 19
	60S ribosomal protein L36a-like (Large ribosomal subunit protein eL42-like)
	Protein PRR14L (Proline rich 14-like protein)
	Translocon-associated protein subunit gamma
**ii.**	**32 common elements in "100 µM c-di-AMP+*Pg* LPS↓↓" and "*Pg* LPS↓↓":**
	CD99 antigen (12E7) (E2 antigen) (Protein MIC2) (T-cell surface glycoprotein E2) (CD antigen CD99)
	Leucine zipper and CTNNBIP1 domain containing; isoform CRA_a
	cDNA FLJ76106; highly similar to Homo sapiens neurolysin (metallopeptidase M3 family) (NLN); mRNA
	40S ribosomal protein S19 (Ribosomal protein S19; isoform CRA_a) (cDNA; FLJ92047; Homo sapiens ribosomal protein S19 (RPS19); mRNA)
	HCG19665; isoform CRA_a
	Protein bicaudal D homolog 2 (Bic-D 2)
	DAZ-associated protein 1
	Adenosine kinase (AK) (EC 2.7.1.20) (Adenosine 5'-phosphotransferase)
	Uncharacterized protein DKFZp781O2021
	Nucleolar RNA helicase 2 (EC 3.6.4.13) (DEAD box protein 21) (Gu-alpha) (Nucleolar RNA helicase Gu) (Nucleolar RNA helicase II) (RH II/Gu)
	Phosphoinositide phospholipase C (EC 3.1.4.11)
	Arginine--tRNA ligase; cytoplasmic (EC 6.1.1.19) (Arginyl-tRNA synthetase) (ArgRS)
	ATP-dependent 6-phosphofructokinase; platelet type (ATP-PFK) (PFK-P) (EC 2.7.1.11) (6-phosphofructokinase type C) (Phosphofructo-1-kinase isozyme C) (PFK-C) (Phosphohexokinase)
	Procollagen-lysine 1, 2-oxoglutarate 5-dioxygenase 1, isoform CRA_a
	Sorting nexin-4
	cDNA FLJ76707; highly similar to Homo sapiens transmembrane protein 87A; mRNA
	Mitotic-spindle organizing protein 2B
	Fibronectin type III domain containing 3A; isoform CRA_b
	IL6ST isoform 4
	cDNA FLJ57657; highly similar to Charged multivesicular body protein 1a
	NFU1 iron-sulfur cluster scaffold homolog; mitochondrial (HIRA-interacting protein 5)
	cDNA FLJ78024
	Gamma-tubulin complex component 3 (GCP-3) (hGCP3) (Gamma-ring complex protein 104 kDa) (h104p) (hGrip104) (Spindle pole body protein Spc98 homolog) (hSpc98)
	Mothers against decapentaplegic homolog (MAD homolog) (Mothers against DPP homolog) (SMAD family member) (Fragment)
	Frequenin homolog (Drosophila); isoform CRA_a

	cDNA FLJ56673; highly similar to Homo sapiens adipocyte-specific adhesion molecule (ASAM); mRNA
	CCR4-NOT transcription complex; subunit 3; isoform CRA_a
	Sorting nexin-27
	Serine/threonine-protein kinase N2 (EC 2.7.11.13) (PKN gamma) (Protein kinase C-like 2) (Protein-kinase C-related kinase 2)
	Large neutral amino acids transporter small subunit 1 (4F2 light chain) (4F2 LC) (4F2LC) (CD98 light chain) (Integral membrane protein E16) (E16) (L-type amino acid transporter 1) (hLAT1) (Solute carrier family 7 member 5) (y+ system cationic amino acid transporter)
	Core-binding factor; beta subunit; isoform CRA_b
	Sushi domain containing 2; isoform CRA_a (Testicular tissue protein Li 190)
**iii.**	**22 common elements in "100 µM c-di-GMP+*Pg* LPS↓↓" and "*Pg* LPS↓↓":**
	Histone H2B
	Glycerol-3-phosphate phosphatase (G3PP) (EC 3.1.3.21) (Aspartate-based ubiquitous Mg(2+)-dependent phosphatase) (AUM) (EC 3.1.3.48) (Phosphoglycolate phosphatase) (PGP)
	Structural maintenance of chromosomes protein
	Pachytene checkpoint protein 2 homolog (Human papillomavirus type 16 E1 protein-binding protein) (16E1-BP) (HPV16 E1 protein-binding protein) (Thyroid hormone receptor interactor 13) (Thyroid receptor-interacting protein 13) (TR-interacting protein 13) (TRIP-13)
	Serine/threonine-protein phosphatase PGAM5; mitochondrial (EC 3.1.3.16) (Bcl-XL-binding protein v68) (Phosphoglycerate mutase family member 5)
	Pescadillo homolog
	Uncharacterized protein CAD (Fragment)
	MRPS9 protein (Fragment)
	Bumetanide-sensitive Na-K-Cl cotransporter (Solute carrier family 12 (Sodium/potassium/chloride transporters); member 2; isoform CRA_b)
	Uncharacterized protein DKFZp547A0616 (Fragment)
	Golgi-associated PDZ and coiled-coil motif-containing protein (CFTR-associated ligand) (Fused in glioblastoma) (PDZ protein interacting specifically with TC10) (PIST)
	Transcriptional enhancer factor TEF-1
	Heterogeneous nuclear ribonucleoprotein L-like
	Nucleoporin Nup37 (p37) (Nup107-160 subcomplex subunit Nup37)
	cDNA; FLJ96555
	Processing of 1; ribonuclease P/MRP subunit (S. cerevisiae)
	cDNA FLJ75682; highly similar to Homo sapiens armadillo repeat containing; X-linked 3 (ARMCX3); transcript variant 1; mRNA
	28S ribosomal protein S17; mitochondrial (Fragment)
	EH domain-binding protein 1-like protein 1
	Ras-related protein Rab-22A (Rab-22)
	cDNA FLJ50983; highly similar to Homo sapiens lysocardiolipin acyltransferase (LYCAT); transcript variant 1; mRNA
	cDNA; FLJ96877

iv.	102 elements included exclusively in "*Pg* LPS↓↓":
	Damage-control phosphatase ARMT1 (EC 3.1.3.-) (Acidic residue methyltransferase 1) (Protein-glutamate O-methyltransferase) (EC 2.1.1.-) (Sugar phosphate phosphatase ARMT1)
	TMEM214 protein (Fragment)
	SLC25A5 protein (Fragment)
	Core histone macro-H2A
	HCG2032701; isoform CRA_a
	Histocompatibility 13 isoform 1 (Fragment)
	Signal recognition particle receptor beta subunit (Signal recognition particle receptor; B subunit; isoform CRA_b)
	cDNA FLJ54042; highly similar to ATP-binding cassette sub-family D member 3
	cDNA FLJ58355; highly similar to Tyrosine-protein phosphatase non-receptor type 23
	Amino acid transporter (Fragment)
	Translocating chain-associated membrane protein 1 (Translocation associated membrane protein 1; isoform CRA_a)
	Stromal cell derived factor 4; isoform CRA_c
	Guanine nucleotide-binding protein G(i) subunit alpha (G(i) alpha-3)
	HCG2024613; isoform CRA_a
	Mitochondrial proton/calcium exchanger protein (Leucine zipper-EF-hand-containing transmembrane protein 1)
	cDNA; FLJ92955; highly similar to Homo sapiens transportin-SR (TRN-SR); mRNA
	Acetolactate synthase-like protein (EC 2.2.1.-) (IlvB-like protein)
	40S ribosomal protein S26
	Stimulator of interferon protein
	Dolichol-phosphate mannosyltransferase subunit 1 (EC 2.4.1.83) (Fragment)
	Surfeit 4 (Surfeit locus protein 4)
	Collagen; type VI; alpha 3 (Epididymis secretory sperm binding protein)
	Protein YIF1B (YIP1-interacting factor homolog B)
	cDNA FLJ56280; highly similar to Endoplasmic reticulum-Golgi intermediate compartment protein 1
	cDNA FLJ90278 fis; clone NT2RP1000325; highly similar to Phosphate carrier protein; mitochondrialprecursor
	Arylacetamide deacetylase-like 1 (Neutral cholesterol ester hydrolase 1)
	Collagen alpha-1(VI) chain
	cDNA FLJ16830 fis; clone UTERU3022536; highly similar to Chromodomain helicase-DNA-binding protein 4
	Copine III; isoform CRA_a
	60S ribosomal protein L26-like 1 (Large ribosomal subunit protein uL24-like 1)
	E3 SUMO-protein ligase RanBP2 (EC 2.3.2.-) (358 kDa nucleoporin) (Nuclear pore complex protein Nup358) (Nucleoporin Nup358) (Ran-binding protein 2) (RanBP2) (p270)
	CD97 antigen (Leukocyte antigen CD97) (CD antigen CD97) [Cleaved into: CD97 antigen subunit alpha; CD97 antigen subunit beta]

Translocase of outer mitochondrial membrane 20 homolog (Yeast); isoform CRA_a
Unc-84 homolog B (C. elegans); isoform CRA_b
ATPase Ca++ transporting cardiac muscle slow twitch 2 isoform 1 (ATPase; Ca++ transporting; cardiac muscle; slow twitch 2; isoform CRA_e) (Fragment)
Leucine rich repeat (In FLII) interacting protein 1; isoform CRA_c (Leucine-rich repeat flightless-interacting protein 1)
Small ubiquitin-related modifier 5 (SUMO-5) (SUMO1 pseudogene 1) (Ubiquitin-like 2) (Ubiquitin-like 6)
Endothelin-converting enzyme 1 (cDNA FLJ59212; highly similar to Endothelin-converting enzyme 1)
Calcium-transporting ATPase (EC 7.2.2.10)
Cell proliferation-inducing protein 47 (HCG39985; isoform CRA_a) (cDNA; FLJ94101; Homo sapiens ATP synthase; H+ transporting; mitochondrial F0complex; subunit b; isoform 1 (ATP5F1); mRNA)
E3 ubiquitin-protein ligase UHRF1 (Ubiquitin-like; containing PHD and RING finger domains; 1; isoform CRA_b)
ABC50 protein (ATP-binding cassette; sub-family F (GCN20); member 1) (ATP-binding cassette; sub-family F (GCN20); member 1; isoform CRA_a)
Succinyl-CoA:3-ketoacid-coenzyme A transferase (EC 2.8.3.5)
NADH dehydrogenase [ubiquinone] iron-sulfur protein 2; mitochondrial (EC 1.6.99.3) (EC 7.1.1.2) (Complex I-49kD) (CI-49kD) (NADH-ubiquinone oxidoreductase 49 kDa subunit)
cDNA FLJ55135; highly similar to Di-N-acetylchitobiase
Mannose-P-dolichol utilization defect 1 protein
39S ribosomal protein L38; mitochondrial (L38mt) (MRP-L38) (Mitochondrial large ribosomal subunit protein mL38)
E3 ubiquitin-protein ligase RNF114 (Fragment)
CGI-111 protein
Tumor necrosis factor receptor type 1-associated DEATH domain protein (TNFR1-associated DEATH domain protein) (TNFRSF1A-associated via death domain)
Chondrosarcoma-associated protein 2
Plasma alpha-L-fucosidase (EC 3.2.1.51) (Alpha-L-fucoside fucohydrolase 2) (Alpha-L-fucosidase 2)
Matrix metalloproteinase 1 preproprotein variant (Fragment)
Mitochondrial ribosomal protein L49; isoform CRA_b
Cytidine 5'-monophosphate N-acetylneuraminic acid synthetase variant (Fragment)
cDNA FLJ75454; highly similar to Homo sapiens arrestin; beta 1 (ARRB1); transcript variant 1; mRNA
Solute carrier family 25 member 4 isoform 1 (Fragment)
WD repeat-containing protein 26
cDNA FLJ56289; highly similar to Homo sapiens sperm specific antigen 2 (SSFA2); mRNA
cDNA FLJ10529 fis; clone NT2RP2000965; highly similar to Targeting protein for Xklp2

Active breakpoint cluster region-related protein (cDNA FLJ54747; highly similar to Active breakpoint cluster region-related protein)
HCG1989366; isoform CRA_a
E3 ubiquitin-protein ligase MARCH5 (EC 2.3.2.27) (Membrane-associated RING finger protein 5) (Membrane-associated RING-CH protein V) (MARCH-V) (Mitochondrial ubiquitin ligase) (MITOL) (RING finger protein 153) (RING-type E3 ubiquitin transferase MARCH5)
cDNA FLJ75083; highly similar to Homo sapiens amine oxidase (flavin containing) domain 2 (AOF2);mRNA
MICOS complex subunit
COMM domain-containing protein 2
Chromosome 10 open reading frame 70; isoform CRA_b
Polio virus related protein 2; alpha isoform (Fragment)
Peptidyl-prolyl cis-trans isomerase (PPIase) (EC 5.2.1.8) (Fragment)
cDNA PSEC0127 fis; clone PLACE1003724; highly similar to Mitochondrial 28S ribosomal protein S22
Replication factor C subunit 2 (Activator 1 40 kDa subunit) (A1 40 kDa subunit) (Activator 1 subunit 2) (Replication factor C 40 kDa subunit) (RF-C 40 kDa subunit) (RFC40)
Golgi transport 1 homolog B (S. cerevisiae); isoform CRA_c (Vesicle transport protein GOT1B)
Retinal rod rhodopsin-sensitive cGMP 3';5'-cyclic phosphodiesterase subunit delta
Mitochondrial peptide methionine sulfoxide reductase (EC 1.8.4.11) (Peptide-methionine (S)-S-oxide reductase) (Peptide Met(O) reductase) (Protein-methionine-S-oxide reductase) (PMSR)
ADP-ribosylation factor GTPase-activating protein 2 (Fragment)
Gasdermin domain containing 1; isoform CRA_d (Gasdermin-D)
Calcium uniporter protein; mitochondrial (HsMCU) (Coiled-coil domain-containing protein 109A)
RNA polymerase II-associated protein 3
Oxysterol-binding protein-related protein 3 (ORP-3) (OSBP-related protein 3)
Ribosome biogenesis regulatory protein homolog
Epidermal growth factor receptor pathway substrate 15-like 1; isoform CRA_a
Ribosome biogenesis protein WDR12 (WD repeat-containing protein 12)
Phosphofurin acidic cluster sorting protein 1; isoform CRA_a
Diacylglycerol kinase (DAG kinase) (EC 2.7.1.107)
NADH dehydrogenase [ubiquinone] 1 beta subcomplex subunit 9
60S ribosomal protein L22-like 1
Importin subunit alpha
Cadherin 2; type 1; N-cadherin (Neuronal); isoform CRA_b
ATP-dependent Clp protease proteolytic subunit; mitochondrial (EC 3.4.21.92) (Endopeptidase Clp)
Heme oxygenase (EC 1.14.14.18)
Transmembrane protein 205 (Fragment)

	Cytochrome b-c1 complex subunit 9 (Complex III subunit 9) (Complex III subunit X) (Cytochrome c1 non-heme 7 kDa protein) (Ubiquinol-cytochrome c reductase complex 7.2 kDa protein)
	Contactin-associated protein 1 (Caspr) (Caspr1) (Neurexin IV) (Neurexin-4) (p190)
	Protocadherin gamma-A9 (PCDH-gamma-A9)
	DNAJC1 protein (Fragment)
	Phosphoinositide phospholipase C (EC 3.1.4.11) (Fragment)
	39S ribosomal protein L13; mitochondrial (L13mt) (MRP-L13) (Mitochondrial large ribosomal subunit protein uL13m)
	Tetratricopeptide repeat protein 9C (Fragment)
	Torsin-1B (Torsin ATPase-1B) (EC 3.6.4.-) (Torsin family 1 member B)
	[F-actin]-monooxygenase MICAL2
	Starch-binding domain-containing protein 1 (Genethonin-1) (Glycophagy cargo receptor STBD1)
	MHC class I antigen (Fragment)
**v.**	**106 elements included exclusively in "100 µM c-di-GMP+*Pg* LPS↓↓":**
	Collagen alpha-1(XV) chain
	Collagen alpha-1(I) chain (Alpha-1 type I collagen)
	cDNA FLJ90619 fis; clone PLACE1002374; highly similar to Cathepsin L
	Beta-glucuronidase (EC 3.2.1.31)
	Retinol dehydrogenase 10 (All-trans); isoform CRA_a
	Collagen; type I; alpha 2; isoform CRA_c (Epididymis secretory sperm binding protein)
	Collagen alpha-1(III) chain
	Epididymis luminal protein 164
	Collagen; type I; alpha 1; isoform CRA_a
	Tropomyosin 1 (Alpha); isoform CRA_a
	Histone H3
	Selenide; water dikinase 1 (EC 2.7.9.3) (Selenium donor protein 1) (Selenophosphate synthase 1)
	Tyrosine 3-monooxygenase/tryptophan 5-monooxygenase activation protein; eta polypeptide; isoform CRA_b
	Thymidylate synthetase (Thymidylate synthetase; isoform CRA_f) (cDNA; FLJ94841; Homo sapiens thymidylate synthetase (TYMS); mRNA)
	Epididymis secretory protein Li 283
	WD repeat-containing protein 44 (Rabphilin-11)
	Glutamate--cysteine ligase regulatory subunit (GCS light chain) (Gamma-ECS regulatory subunit) (Gamma-glutamylcysteine synthetase regulatory subunit) (Glutamate--cysteine ligase modifier subunit)
	Centrin-2 (Caltractin isoform 1)
	Uveal autoantigen with coiled-coil domains and ankyrin repeats
	ERBB2IP protein
	1-phosphatidylinositol 4;5-bisphosphate phosphodiesterase beta-4 (EC 3.1.4.11) (Phosphoinositide phospholipase C-beta-4) (Phospholipase C-beta-4) (PLC-beta-4)

	UDP-N-acetylhexosamine pyrophosphorylase (Antigen X) (AGX) (Sperm-associated antigen 2) [Includes: UDP-N-acetylgalactosamine pyrophosphorylase (EC 2.7.7.83) (AGX-1); UDP-N-acetylglucosamine pyrophosphorylase (EC 2.7.7.23) (AGX-2)]
	La-related protein 1 (La ribonucleoprotein domain family member 1)
	MRPL1 protein (Fragment)
	Ubiquitin-like modifier-activating enzyme 5
	Arylsulfatase B; isoform CRA_a
	Protein O-GlcNAcase (OGA) (EC 3.2.1.169) (Beta-N-acetylglucosaminidase) (Beta-N-acetylhexosaminidase) (Beta-hexosaminidase) (Meningioma-expressed antigen 5) (N-acetyl-beta-D-glucosaminidase) (N-acetyl-beta-glucosaminidase) (Nuclear cytoplasmic O-GlcNAcase and acetyltransferase) (NCOAT)
	Multifunctional fusion protein [Includes: L-glutamate gamma-semialdehyde dehydrogenase (EC 1.2.1.88) (L-glutamate gamma-semialdehyde dehydrogenase); Delta-1-pyrroline-5-carboxylate dehydrogenase (P5C dehydrogenase)]
	cDNA FLJ54090; highly similar to 4F2 cell-surface antigen heavy chain
	Ezrin
	HEXA protein (Fragment)
	Arginine/serine-rich splicing factor 6 variant (Fragment)
	Replication protein A3; 14kDa (Replication protein A3; 14kDa; isoform CRA_b) (cDNA; FLJ92105; Homo sapiens replication protein A3; 14kDa (RPA3); mRNA)
	28S ribosomal protein S31; mitochondrial (MRP-S31) (S31mt) (Imogen 38) (Mitochondrial small ribosomal subunit protein mS31)
	Malic enzyme
	Uncharacterized protein DKFZp686M0619 (Fragment)
	DNA topoisomerase 2-alpha (EC 5.6.2.2) (DNA topoisomerase II; alpha isozyme)
	Dehydrogenase/reductase SDR family member 4 (EC 1.1.1.184) (NADPH-dependent carbonyl reductase/NADP-retinol dehydrogenase) (CR) (PHCR) (NADPH-dependent retinol dehydrogenase/reductase) (NRDR) (humNRDR) (Peroxisomal short-chain alcohol dehydrogenase) (PSCD) (SCAD-SRL) (Short chain dehydrogenase/reductase family 25C member 2) (Short-chain dehydrogenase/reductase family member 4)
	Ribosome biogenesis protein BOP1 (Block of proliferation 1 protein)
	Inositol-1-monophosphatase (EC 3.1.3.25)
	Stathmin (Fragment)
	Monofunctional C1-tetrahydrofolate synthase; mitochondrial
	Nesprin-1 (Enaptin) (KASH domain-containing protein 1) (KASH1) (Myocyte nuclear envelope protein 1) (Myne-1) (Nuclear envelope spectrin repeat protein 1) (Synaptic nuclear envelope protein 1) (Syne-1)
	Trafficking protein particle complex subunit
	DNA helicase (EC 3.6.4.12)
	NADPH:adrenodoxin oxidoreductase; mitochondrial (EC 1.18.1.6)
	N-alpha-acetyltransferase 15; NatA auxiliary subunit
	Quinone oxidoreductase (EC 1.6.5.5) (NADPH:quinone reductase) (Zeta-crystallin)
	cDNA FLJ75877; highly similar to Homo sapiens 5'-nucleotidase; cytosolic II (NT5C2); mRNA
	cDNA FLJ54257; highly similar to Leiomodin-1

	DNA-directed RNA polymerase subunit beta (EC 2.7.7.6)
	DNA helicase (EC 3.6.4.12) (Fragment)
	Membrane-associated progesterone receptor component 2 (Progesterone membrane-binding protein) (Steroid receptor protein DG6)
	Nestin; isoform CRA_c
	Dipeptidylpeptidase III isoform 1 variant (Fragment)
	Protein SET
	Sister chromatid cohesion protein PDS5 homolog B (Androgen-induced proliferation inhibitor) (Androgen-induced prostate proliferative shutoff-associated protein AS3)
	Plectin (PCN) (PLTN) (Hemidesmosomal protein 1) (HD1) (Plectin-1)
	Family with sequence similarity 62 (C2 domain containing); member A; isoform CRA_a
	Protein NipSnap homolog 3A (NipSnap3A) (Protein NipSnap homolog 4) (NipSnap4) (Target for Salmonella secreted protein C) (TassC)
	NUMA1 variant protein (Fragment)
	Myoferlin (Fer-1-like protein 3)
	cDNA FLJ53963; highly similar to Leukocyte elastase inhibitor
	Splicing factor 3B subunit 3 (Pre-mRNA-splicing factor SF3b 130 kDa subunit) (SF3b130) (STAF130) (Spliceosome-associated protein 130) (SAP 130)
	Spectrin beta chain
	DNA replication licensing factor MCM6 (EC 3.6.4.12) (p105MCM)
	Lamin-B1
	Collagen alpha-1(XII) chain
	EH-domain containing 2; isoform CRA_a
	Unconventional myosin-Ib
	Casein kinase II subunit alpha
	U5 small nuclear ribonucleoprotein 200 kDa helicase (EC 3.6.4.13) (Activating signal cointegrator 1 complex subunit 3-like 1) (BRR2 homolog) (U5 snRNP-specific 200 kDa protein) (U5-200KD)
	HCG1991735; isoform CRA_a
	Band 4.1-like protein 2 (Generally expressed protein 4.1) (4.1G)
	Unconventional myosin-Ic
	Sulfatase modifying factor 2 isoform 2
	Rho guanine nucleotide exchange factor 10 (Fragment)
	Phosphorylated adapter RNA export protein (RNA U small nuclear RNA export adapter protein)
	Carboxymethylenebutenolidase homolog (EC 3.1.-.-)
	cDNA FLJ58014; highly similar to Homo sapiens programmed cell death 4; transcript variant 1; mRNA
	Ribosome production factor 2 homolog (Fragment)
	Kinesin-like protein KIF20A (GG10_2) (Mitotic kinesin-like protein 2) (MKlp2) (Rab6-interacting kinesin-like protein) (Rabkinesin-6)
	Protein phosphatase 1; regulatory (Inhibitor) subunit 8

	Tyrosine-protein kinase BAZ1B (EC 2.7.10.2) (Bromodomain adjacent to zinc finger domain protein 1B) (Williams syndrome transcription factor) (Williams-Beuren syndrome chromosomal region 10 protein) (Williams-Beuren syndrome chromosomal region 9 protein) (hWALp2)
	Apolipoprotein B mRNA editing enzyme catalytic polypeptide-like 3B (Apolipoprotein B mRNA editing enzyme; catalytic polypeptide-like 3B; isoform CRA_c)
	ADP-ribosylation factor-like protein 6-interacting protein 4 (Fragment)
	Torsin family 1; member A (Torsin A); isoform CRA_a (cDNA FLJ56343; highly similar to Torsin A)
	Multivesicular body subunit 12A
	CREB1 protein (cAMP responsive element binding protein 1) (cAMP responsive element binding protein 1; isoform CRA_b) (cDNA; FLJ96224; Homo sapiens cAMP responsive element binding protein 1 (CREB1);transcript variant A; mRNA) (Fragment)
	Argininosuccinate lyase isoform 1 (Argininosuccinate lyase; isoform CRA_b)
	DNA-directed RNA polymerases I; II; and III subunit RPABC1
	Activating signal cointegrator 1 complex subunit 3 (EC 3.6.4.12) (ASC-1 complex subunit p200) (ASC1p200) (Helicase; ATP binding 1) (Trip4 complex subunit p200)
	Heat shock 27kDa protein family; member 7 (Cardiovascular); isoform CRA_c (Heat shock protein beta-7) (cDNA FLJ34956 fis; clone NTONG2003158; highly similar to Heat shock 27kD protein family; member 7)
	Palmitoyl-protein thioesterase 1
	FLJ00144 protein (Fragment)
	V-type proton ATPase 16 kDa proteolipid subunit (V-ATPase 16 kDa proteolipid subunit) (Vacuolar proton pump 16 kDa proteolipid subunit)
	EF-hand domain-containing protein D1 (EF-hand domain-containing protein 1) (Swiprosin-2)
	U6 snRNA-associated Sm-like protein LSm7
	NADH dehydrogenase (Ubiquinone) 1 alpha subcomplex; 4; 9kDa; isoform CRA_b
	Beta-4 tubulin (Fragment)
	High mobility group AT-hook 1
	Microtubule-associated proteins 1A/1B light chain 3A (Autophagy-related protein LC3 A) (Autophagy-related ubiquitin-like modifier LC3 A) (MAP1 light chain 3-like protein 1) (MAP1A/MAP1B light chain 3 A) (MAP1A/MAP1B LC3 A) (Microtubule-associated protein 1 light chain 3 alpha)
	cDNA FLJ58024; highly similar to NADH-ubiquinone oxidoreductase 20 kDa subunit; mitochondrial
	Ribulose-phosphate 3-epimerase (EC 5.1.3.1) (Ribulose-5-phosphate-3-epimerase)
	Protein C10
	Chromosome-associated kinesin KIF4A (Chromokinesin-A)
**vi.**	**115 elements included exclusively in "100 µM c-di-AMP+*Pg* LPS↓↓":**
	Phosphoserine phosphatase (Fragment)
	Putative 60S ribosomal protein L39-like 5 (60S ribosomal protein L39 pseudogene 5)
	Ribosomal protein L38; isoform CRA_a

SH2 domain-containing protein 4A (Protein SH(2)A) (Protein phosphatase 1 regulatory subunit 38)
cDNA FLJ76855; highly similar to Homo sapiens exportin 7 (XPO7); mRNA
Ubiquitin-fold modifier-conjugating enzyme 1 (Ufm1-conjugating enzyme 1)
40S ribosomal protein S5 (Fragment)
Coatomer subunit gamma
Protein S100-A6 (Calcyclin) (Growth factor-inducible protein 2A9) (MLN 4) (Prolactin receptor-associated protein) (PRA) (S100 calcium-binding protein A6)
DCC-interacting protein 13-beta (Dip13-beta) (Adapter protein containing PH domain; PTB domain and leucine zipper motif 2)
60S ribosomal protein L18a
Phosphoribosyl pyrophosphate synthetase-associated protein 1; isoform CRA_a
Dr1-associated corepressor
Ribonucloprotein
Proteasome subunit beta type-5 (EC 3.4.25.1) (Macropain epsilon chain) (Multicatalytic endopeptidase complex epsilon chain) (Proteasome chain 6) (Proteasome epsilon chain) (Proteasome subunit MB1) (Proteasome subunit X)
Farnesyl pyrophosphate synthase (FPP synthase) (FPS) (EC 2.5.1.10) ((2E;6E)-farnesyl diphosphate synthase) (Dimethylallyltranstransferase) (EC 2.5.1.1) (Farnesyl diphosphate synthase) (Geranyltranstransferase)
cDNA FLJ76072; highly similar to Homo sapiens GIPC PDZ domain containing family; member 1 (GIPC1); transcript variant 1; mRNA
Phosphoserine aminotransferase (EC 2.6.1.52)
Pyruvate kinase (EC 2.7.1.40)
Tyrosine--tRNA ligase (EC 6.1.1.1) (Tyrosyl-tRNA synthetase) (Fragment)
Ribosomal protein L7; isoform CRA_a
Ras-related protein R-Ras2 (Ras-like protein TC21) (Teratocarcinoma oncogene)
Squamous cell carcinoma antigen recognized by T-cells 3
Basic leucine zipper and W2 domains 1, isoform CRA_a
40S ribosomal protein S15
V-type proton ATPase subunit C
Rho-associated protein kinase (EC 2.7.11.1)
Alanine--tRNA ligase; cytoplasmic (EC 6.1.1.7) (Alanyl-tRNA synthetase) (AlaRS) (Renal carcinoma antigen NY-REN-42)
cDNA FLJ54534; highly similar to Homo sapiens cysteinyl-tRNA synthetase (CARS); transcript variant 3; mRNA
Signal recognition particle subunit SRP68 (SRP68) (Signal recognition particle 68 kDa protein)
Eukaryotic translation initiation factor 3 subunit C (eIF3c) (Eukaryotic translation initiation factor 3 subunit 8) (eIF3 p110)
Heat shock protein 90kDa alpha (Cytosolic); class B member 1; isoform CRA_a
cDNA FLJ12454 fis; clone NT2RM1000555; highly similar to UNR PROTEIN (cDNA FLJ12466 fis; clone NT2RM1000826; highly similar to UNR PROTEIN)
Actinin alpha 4 isoform 1 (Fragment)
Fascin

High density lipoprotein binding protein (Vigilin); isoform CRA_a (Vigilin)
Programmed cell death 6-interacting protein (PDCD6-interacting protein) (ALG-2-interacting protein 1) (ALG-2-interacting protein X) (Hp95)
Rab GDP dissociation inhibitor
Signal recognition particle subunit SRP72
Glutamine--fructose-6-phosphate aminotransferase [isomerizing] 1 (EC 2.6.1.16) (D-fructose-6-phosphate amidotransferase 1) (Glutamine:fructose-6-phosphate amidotransferase 1) (GFAT 1) (GFAT1) (Hexosephosphate aminotransferase 1)
L-lactate dehydrogenase (EC 1.1.1.27)
Proteasome subunit beta (EC 3.4.25.1) (Fragment)
40S ribosomal protein S3 (EC 4.2.99.18) (Small ribosomal subunit protein uS3)
Aspartate aminotransferase (EC 2.6.1.1)
Glucose-6-phosphate 1-dehydrogenase (EC 1.1.1.49)
Glycyl-tRNA synthetase
Thrombospondin-1 (Glycoprotein G)
Serine/threonine-protein phosphatase 2A 56 kDa regulatory subunit
DDX39B (HCG2005638; isoform CRA_a)
Eukaryotic translation initiation factor 3 subunit L (eIF3l) (Eukaryotic translation initiation factor 3 subunit 6-interacting protein) (Eukaryotic translation initiation factor 3 subunit E-interacting protein)
Nuclear autoantigenic sperm protein (NASP)
ATP-citrate synthase (EC 2.3.3.8) (ATP-citrate (pro-S-)-lyase) (Citrate cleavage enzyme)
Phosphatidylinositol transfer protein beta isoform (PI-TP-beta) (PtdIns transfer protein beta) (PtdInsTP beta)
Vacuolar protein sorting-associated protein 35 (hVPS35) (Maternal-embryonic 3) (Vesicle protein sorting 35)
Eukaryotic translation initiation factor 5A-1 (eIF-5A-1) (eIF-5A1) (Eukaryotic initiation factor 5A isoform 1) (eIF-5A) (Rev-binding factor) (eIF-4D)
Clathrin heavy chain
60S ribosomal protein L3 (HIV-1 TAR RNA-binding protein B) (TARBP-B) (Large ribosomal subunit protein uL3)
N-acylaminoacyl-peptide hydrolase, isoform CRA_b
Proliferation-associated 2G4; 38kDa; isoform CRA_a
Heterogeneous nuclear ribonucleoprotein Q (hnRNP Q) (Glycine- and tyrosine-rich RNA-binding protein) (GRY-RBP) (NS1-associated protein 1) (Synaptotagmin-binding; cytoplasmic RNA-interacting protein)
Glucan; branching enzyme 1 variant (Fragment)
26S proteasome non-ATPase regulatory subunit 1
Testicular tissue protein Li 192
Mitotic checkpoint protein BUB3 (Fragment)
Eukaryotic translation initiation factor 3 subunit A (eIF3a) (Eukaryotic translation initiation factor 3 subunit 10) (eIF-3-theta)
Pumilio homolog 1 (Fragment)
Bifunctional glutamate/proline--tRNA ligase (Bifunctional aminoacyl-tRNA synthetase) (Cell proliferation-inducing gene 32 protein) (Glutamatyl-prolyl-tRNA

synthetase) [Includes: Glutamate--tRNA ligase (EC 6.1.1.17) (Glutamyl-tRNA synthetase) (GluRS); Proline--tRNA ligase (EC 6.1.1.15) (Prolyl-tRNA synthetase)]
Tubulin alpha-1B chain (Alpha-tubulin ubiquitous) (Tubulin K-alpha-1) (Tubulin alpha-ubiquitous chain) [Cleaved into: Detyrosinated tubulin alpha-1B chain]
Testicular secretory protein Li 63 (Ubiquitin-activating enzyme E1 (A1S9T and BN75 temperature sensitivity complementing); isoform CRA_a)
N-alpha-acetyltransferase 10 (EC 2.3.1.255) (N-terminal acetyltransferase complex ARD1 subunit homolog A) (hARD1) (NatA catalytic subunit Naa10)
Catenin (Cadherin-associated protein); alpha 1; 102kDa; isoform CRA_b (Epididymis secretory sperm binding protein)
Asparagine--tRNA ligase; cytoplasmic (EC 6.1.1.22) (Asparaginyl-tRNA synthetase) (AsnRS) (Asparaginyl-tRNA synthetase 1)
Spermidine synthase (SPDSY) (EC 2.5.1.16) (Putrescine aminopropyltransferase)
Kinesin-like protein
Epididymis luminal protein 33 (Epididymis secretory sperm binding protein) (Epididymis secretory sperm binding protein Li 72p) (Heat shock 70kDa protein 8; isoform CRA_a)
ARP3 actin-related protein 3 homolog (Yeast); isoform CRA_a
Ribosome assembly factor mrt4
DNA damage-binding protein 1 (DDB p127 subunit) (DNA damage-binding protein a) (DDBa) (Damage-specific DNA-binding protein 1) (HBV X-associated protein 1) (XAP-1) (UV-damaged DNA-binding factor) (UV-damaged DNA-binding protein 1) (UV-DDB 1) (XPE-binding factor) (XPE-BF) (Xeroderma pigmentosum group E-complementing protein) (XPCe)
Testis secretory sperm-binding protein Li 197a
SEC31-like 1 (S. cerevisiae), isoform CRA_d
Seryl-tRNA synthetase variant (Fragment)
Vacuolar protein sorting-associated protein 26A (Vesicle protein sorting 26A) (hVPS26)
Epididymis luminal protein 70 (Moesin; isoform CRA_a)
Queuosine salvage protein
Putative hydroxypyruvate isomerase (Fragment)
Bax inhibitor 1 (Fragment)
RWD domain-containing protein 1 (Fragment)
cDNA FLJ58285; highly similar to Homo sapiens pre-B-cell leukemia transcription factor interacting protein 1 (PBXIP1); mRNA
cDNA FLJ12434 fis; clone NT2RM1000037; highly similar to Homo sapiens KIAA0690 protein
Exocyst complex component
Echinoderm microtubule-associated protein-like 1
cDNA FLJ50791; highly similar to Nitrilase homolog 1
Ectopic P granules protein 5 homolog
E3 ubiquitin-protein ligase KCMF1 (EC 2.3.2.27) (FGF-induced in gastric cancer) (Potassium channel modulatory factor) (PCMF) (RING-type E3 ubiquitin transferase KCMF1) (ZZ-type zinc finger-containing protein 1)
Ras-related GTP binding C; isoform CRA_a

	MKI67 FHA domain-interacting nucleolar phosphoprotein (Nucleolar phosphoprotein Nopp34) (Nucleolar protein interacting with the FHA domain of pKI-67) (hNIFK)
	LisH domain-containing protein ARMC9
	Anoctamin-6 (Small-conductance calcium-activated nonselective cation channel) (SCAN channel) (Transmembrane protein 16F)
	Ubiquitin-conjugating enzyme E2 A
	cDNA FLJ51512; highly similar to Periodic tryptophan protein 1 homolog
	Protein diaphanous homolog 3 (Diaphanous-related formin-3) (DRF3) (MDia2)
	Esterase OVCA2 (EC 3.1.2.-) (Ovarian cancer-associated gene 2 protein)
	Coatomer subunit zeta-2 (Zeta-2-coat protein) (Zeta-2 COP)
	Cyclin-dependent kinases regulatory subunit
	Exocyst complex component 8 (Exocyst complex 84 kDa subunit)
	V-type proton ATPase subunit a
	Integrin alpha-2
	LIM and senescent cell antigen-like-containing domain protein 1 (Particularly interesting new Cys-His protein 1) (PINCH-1) (Renal carcinoma antigen NY-REN-48)
	Solute carrier family 38; member 2; isoform CRA_b
	GL009
	C-Mpl binding protein (La-related protein 4)
	cDNA FLJ57941; highly similar to Nucleoside diphosphate-linked moiety X motif16
	Lysophospholipid acyltransferase 7 (Fragment)
	Peripheral plasma membrane protein CASK
	Protein disulfide-isomerase A3 (Fragment)
**vii.**	**18 common elements in "100 µM c-di-AMP+*Pg* LPS↓↓" and "100 µM c-di-GMP+*Pg* LPS↓↓":**
	DNA mismatch repair protein (Fragment)
	GTPBP4 protein (Fragment)
	Asparagine synthetase [glutamine-hydrolyzing] (EC 6.3.5.4) (Cell cycle control protein TS11) (Glutamine-dependent asparagine synthetase)
	Nuclear pore complex protein Nup88 (88 kDa nucleoporin) (Nucleoporin Nup88)
	IQ motif containing GTPase activating protein 3
	Ribonucleoside-diphosphate reductase (EC 1.17.4.1)
	Proliferating cell nuclear antigen (PCNA) (Cyclin)
	Coronin (Fragment)
	Cathepsin Z (EC 3.4.18.1) (Cathepsin P) (Cathepsin X)
	Fragile X mental retardation syndrome-related protein 1 (cDNA FLJ58644; highly similar to Fragile X mental retardation syndrome-related protein 1)
	Eukaryotic translation initiation factor 3 subunit B (eIF3b) (Eukaryotic translation initiation factor 3 subunit 9) (eIF-3-eta)
	Epididymis luminal protein 4 (Epididymis secretory protein Li 3) (Epididymis secretory protein Li 93) (Tyrosine 3-monooxygenase/tryptophan 5-monooxygenase activation protein zeta polypeptide) (Tyrosine 3-monooxygenase/tryptophan 5-monooxygenase activation protein; zeta polypeptide; isoform CRA_a)

PACSIN2 protein
26S proteasome regulatory subunit 10B
Dihydrofolate reductase (EC 1.5.1.3) (cDNA; FLJ93028; Homo sapiens dihydrofolate reductase (DHFR); mRNA)
RNA-binding protein fox-1 homolog 2 (Fragment)
N(4)-(beta-N-acetylglucosaminyl)-L-asparaginase (EC 3.5.1.26) (Aspartylglucosaminidase) (Glycosylasparaginase) (N4-(N-acetyl-beta-glucosaminyl)-L-asparagine amidase) [Cleaved into: Glycosylasparaginase alpha chain; Glycosylasparaginase beta chain]
U6 snRNA-associated Sm-like protein LSm5
