# Supplementary material for: Activation of cellular responses by cyclic dinucleotides and porphyromonas gingivalis lipopolysaccharide: a proteomic study on gingival fibroblasts
Source: J Oral Microbiol. 2024 Dec 9;17(1):2431453. doi: 10.1080/20002297.2024.2431453 (PMC11632945; doi:10.1080/20002297.2024.2431453)
Supplement: Table_S3_Down_regulated_proteins.pdf [file ZJOM_A_2431453_SM5362.pdf]

**Table S3.** List of downregulated proteins and their distribution in different treatment groups (100 µM c-di-AMP+*Pg* LPS or c-di-GMP+ *Pg* LPS or *Pg* LPS)

| S/No | Treatment/Venn Diagram group |
|---|---|
| **i.** | **8 common elements in "100 µM c-di-AMP+*Pg* LPS↓", "100 µM c-di-GMP+*Pg* LPS↓" and "*Pg* LPS only↓":** |
| | Protein kinase |
| | cDNA FLJ77424; highly similar to Homo sapiens anillin; actin binding protein (scraps homolog; Drosophila); mRNA |
| | TIP41-like protein (Putative MAPK-activating protein PM10) (Type 2A-interacting protein) (TIP) |
| | Threonyl-tRNA synthetase variant (Fragment) |
| | Epididymis secretory sperm binding protein |
| | cDNA; FLJ93949; highly similar to Homo sapiens NIMA (never in mitosis gene a)-related kinase 7 (NEK7); mRNA |
| | G1 to S phase transition 1 |
| | Alpha-parvin (Actopaxin) (CH-ILKBP) (Calponin-like integrin-linked kinase-binding protein) (Matrix-remodeling-associated protein 2) |
| **ii.** | **13 common elements in "100 µM c-di-AMP+*Pg* LPS↓" and "*Pg* LPS only↓":** |
| | CD99 antigen (12E7) (E2 antigen) (Protein MIC2) (T-cell surface glycoprotein E2) (CD antigen CD99) |
| | Leucine zipper and CTNNBIP1 domain containing; isoform CRA_a |
| | cDNA FLJ76106; highly similar to Homo sapiens neurolysin (metallopeptidase M3 family) (NLN); mRNA |
| | 40S ribosomal protein S19 (Ribosomal protein S19; isoform CRA_a) (cDNA; FLJ92047; Homo sapiens ribosomal protein S19 (RPS19); mRNA) |
| | HCG19665; isoform CRA_a |
| | DAZ-associated protein 1 |
| | Adenosine kinase (AK) (EC 2.7.1.20) (Adenosine 5'-phosphotransferase) |
| | Uncharacterized protein DKFZp781O2021 |
| | Nucleolar RNA helicase 2 (EC 3.6.4.13) (DEAD box protein 21) (Gu-alpha) (Nucleolar RNA helicase Gu) (Nucleolar RNA helicase II) (RH II/Gu) |
| | Phosphoinositide phospholipase C (EC 3.1.4.11) |
| | Arginine--tRNA ligase; cytoplasmic (EC 6.1.1.19) (Arginyl-tRNA synthetase) (ArgRS) |
| | ATP-dependent 6-phosphofructokinase; platelet type (ATP-PFK) (PFK-P) (EC 2.7.1.11) (6-phosphofructokinase type C) (Phosphofructo-1-kinase isozyme C) (PFK-C) (Phosphohexokinase) |
| | Procollagen-lysine 1, 2-oxoglutarate 5-dioxygenase 1, isoform CRA_a |
| **iii.** | **15 common elements in "100 µM c-di-AMP+*Pg* LPS↓" and "100 µM c-di-GMP+*Pg* LPS↓":** |
| | DNA mismatch repair protein (Fragment) |
| | TBC1 domain family member 23 (HCV non-structural protein 4A-transactivated protein 1) |

| | |
|---|---|
| | GTPBP4 protein (Fragment) |
| | Asparagine synthetase [glutamine-hydrolyzing] (EC 6.3.5.4) (Cell cycle control protein TS11) (Glutamine-dependent asparagine synthetase) |
| | Nuclear pore complex protein Nup88 (88 kDa nucleoporin) (Nucleoporin Nup88) |
| | IQ motif containing GTPase activating protein 3 |
| | Ribonucleoside-diphosphate reductase (EC 1.17.4.1) |
| | Proliferating cell nuclear antigen (PCNA) (Cyclin) |
| | Coronin (Fragment) |
| | Cathepsin Z (EC 3.4.18.1) (Cathepsin P) (Cathepsin X) |
| | Fragile X mental retardation syndrome-related protein 1 (cDNA FLJ58644; highly similar to Fragile X mental retardation syndrome-related protein 1) |
| | Eukaryotic translation initiation factor 3 subunit B (eIF3b) (Eukaryotic translation initiation factor 3 subunit 9) (eIF-3-eta) |
| | Epididymis luminal protein 4 (Epididymis secretory protein Li 3) (Epididymis secretory protein Li 93) (Tyrosine 3-monooxygenase/tryptophan 5-monooxygenase activation protein zeta polypeptide) (Tyrosine 3-monooxygenase/tryptophan 5-monooxygenase activation protein; zeta polypeptide; isoform CRA_a) |
| | PACSIN2 protein |
| | 26S proteasome regulatory subunit 10B |
| **iv.** | **6 common elements in "100 µM c-di-GMP+*Pg* LPS↓" and "*Pg* LPS only↓":** |
| | Histone H2B |
| | Structural maintenance of chromosomes protein |
| | Pachytene checkpoint protein 2 homolog (Human papillomavirus type 16 E1 protein-binding protein) (16E1-BP) (HPV16 E1 protein-binding protein) (Thyroid hormone receptor interactor 13) (Thyroid receptor-interacting protein 13) (TR-interacting protein 13) (TRIP-13) |
| | Serine/threonine-protein phosphatase PGAM5; mitochondrial (EC 3.1.3.16) (Bcl-XL-binding protein v68) (Phosphoglycerate mutase family member 5) |
| | Pescadillo homolog |
| | Uncharacterized protein CAD (Fragment) |
| **v.** | **45 elements included exclusively in "*Pg* LPS only↓":** |
| | Damage-control phosphatase ARMT1 (EC 3.1.3.-) (Acidic residue methyltransferase 1) (Protein-glutamate O-methyltransferase) (EC 2.1.1.-) (Sugar phosphate phosphatase ARMT1) |
| | TMEM214 protein (Fragment) |
| | SLC25A5 protein (Fragment) |
| | Core histone macro-H2A |
| | HCG2032701; isoform CRA_a |
| | Histocompatibility 13 isoform 1 (Fragment) |
| | Signal recognition particle receptor beta subunit (Signal recognition particle receptor; B subunit; isoform CRA_b) |
| | cDNA FLJ54042; highly similar to ATP-binding cassette sub-family D member 3 |
| | cDNA FLJ58355; highly similar to Tyrosine-protein phosphatase non-receptor type 23 |

| |
|---|
| Amino acid transporter (Fragment) |
| Translocating chain-associated membrane protein 1 (Translocation associated membrane protein 1; isoform CRA_a) |
| Stromal cell derived factor 4; isoform CRA_c |
| Guanine nucleotide-binding protein G(i) subunit alpha (G(i) alpha-3) |
| HCG2024613; isoform CRA_a |
| Mitochondrial proton/calcium exchanger protein (Leucine zipper-EF-hand-containing transmembrane protein 1) |
| cDNA; FLJ92955; highly similar to Homo sapiens transportin-SR (TRN-SR); mRNA |
| Acetolactate synthase-like protein (EC 2.2.1.-) (IlvB-like protein) |
| 40S ribosomal protein S26 |
| Stimulator of interferon protein |
| Dolichol-phosphate mannosyltransferase subunit 1 (EC 2.4.1.83) (Fragment) |
| Surfeit 4 (Surfeit locus protein 4) |
| Collagen; type VI; alpha 3 (Epididymis secretory sperm binding protein) |
| Protein YIF1B (YIP1-interacting factor homolog B) |
| cDNA FLJ56280; highly similar to Endoplasmic reticulum-Golgi intermediate compartment protein 1 |
| cDNA FLJ90278 fis; clone NT2RP1000325; highly similar to Phosphate carrier protein; mitochondrialprecursor |
| Arylacetamide deacetylase-like 1 (Neutral cholesterol ester hydrolase 1) |
| Collagen alpha-1(VI) chain |
| cDNA FLJ16830 fis; clone UTERU3022536; highly similar to Chromodomain helicase-DNA-binding protein 4 |
| Copine III; isoform CRA_a |
| 60S ribosomal protein L26-like 1 (Large ribosomal subunit protein uL24-like 1) |
| E3 SUMO-protein ligase RanBP2 (EC 2.3.2.-) (358 kDa nucleoporin) (Nuclear pore complex protein Nup358) (Nucleoporin Nup358) (Ran-binding protein 2) (RanBP2) (p270) |
| CD97 antigen (Leukocyte antigen CD97) (CD antigen CD97) [Cleaved into: CD97 antigen subunit alpha; CD97 antigen subunit beta] |
| Translocase of outer mitochondrial membrane 20 homolog (Yeast); isoform CRA_a |
| Nucleoporin Nup37 (p37) (Nup107-160 subcomplex subunit Nup37) |
| Unc-84 homolog B (C. elegans); isoform CRA_b |
| ATPase Ca++ transporting cardiac muscle slow twitch 2 isoform 1 (ATPase; Ca++ transporting; cardiac muscle; slow twitch 2; isoform CRA_e) (Fragment) |
| Leucine rich repeat (In FLII) interacting protein 1; isoform CRA_c (Leucine-rich repeat flightless-interacting protein 1) |
| Small ubiquitin-related modifier 5 (SUMO-5) (SUMO1 pseudogene 1) (Ubiquitin-like 2) (Ubiquitin-like 6) |
| Endothelin-converting enzyme 1 (cDNA FLJ59212; highly similar to Endothelin-converting enzyme 1) |
| Calcium-transporting ATPase (EC 7.2.2.10) |

Cell proliferation-inducing protein 47 (HCG39985; isoform CRA_a) (cDNA; FLJ94101; Homo sapiens ATP synthase; H+ transporting; mitochondrial F0complex; subunit b; isoform 1 (ATP5F1); mRNA)

E3 ubiquitin-protein ligase UHRF1 (Ubiquitin-like; containing PHD and RING finger domains; 1; isoform CRA_b)

ABC50 protein (ATP-binding cassette; sub-family F (GCN20); member 1) (ATP-binding cassette; sub-family F (GCN20); member 1; isoform CRA_a)

Succinyl-CoA:3-ketoacid-coenzyme A transferase (EC 2.8.3.5)

NADH dehydrogenase [ubiquinone] iron-sulfur protein 2; mitochondrial (EC 1.6.99.3) (EC 7.1.1.2) (Complex I-49kD) (CI-49kD) (NADH-ubiquinone oxidoreductase 49 kDa subunit)

**vi. 79 elements included exclusively in "100 µM c-di-GMP+*Pg* LPS↓":**

Collagen alpha-1(XV) chain

Collagen alpha-1(I) chain (Alpha-1 type I collagen)

cDNA FLJ90619 fis; clone PLACE1002374; highly similar to Cathepsin L

Beta-glucuronidase (EC 3.2.1.31)

Retinol dehydrogenase 10 (All-trans); isoform CRA_a

Collagen; type I; alpha 2; isoform CRA_c (Epididymis secretory sperm binding protein)

Collagen alpha-1(III) chain

Epididymis luminal protein 164

Collagen; type I; alpha 1; isoform CRA_a

Tropomyosin 1 (Alpha); isoform CRA_a

Histone H3

Selenide; water dikinase 1 (EC 2.7.9.3) (Selenium donor protein 1) (Selenophosphate synthase 1)

Tyrosine 3-monooxygenase/tryptophan 5-monooxygenase activation protein; eta polypeptide; isoform CRA_b

Thymidylate synthetase (Thymidylate synthetase; isoform CRA_f) (cDNA; FLJ94841; Homo sapiens thymidylate synthetase (TYMS); mRNA)

Epididymis secretory protein Li 283

Glycerol-3-phosphate phosphatase (G3PP) (EC 3.1.3.21) (Aspartate-based ubiquitous Mg(2+)-dependent phosphatase) (AUM) (EC 3.1.3.48) (Phosphoglycolate phosphatase) (PGP)

WD repeat-containing protein 44 (Rabphilin-11)

Glutamate--cysteine ligase regulatory subunit (GCS light chain) (Gamma-ECS regulatory subunit) (Gamma-glutamylcysteine synthetase regulatory subunit) (Glutamate--cysteine ligase modifier subunit)

Centrin-2 (Caltractin isoform 1)

Uveal autoantigen with coiled-coil domains and ankyrin repeats

ERBB2IP protein

1-phosphatidylinositol 4;5-bisphosphate phosphodiesterase beta-4 (EC 3.1.4.11) (Phosphoinositide phospholipase C-beta-4) (Phospholipase C-beta-4) (PLC-beta-4)

UDP-N-acetylhexosamine pyrophosphorylase (Antigen X) (AGX) (Sperm-associated antigen 2) [Includes: UDP-N-acetylgalactosamine pyrophosphorylase

| |
|---|
| (EC 2.7.7.83) (AGX-1); UDP-N-acetylglucosamine pyrophosphorylase (EC 2.7.7.23) (AGX-2)] |
| La-related protein 1 (La ribonucleoprotein domain family member 1) |
| MRPL1 protein (Fragment) |
| Ubiquitin-like modifier-activating enzyme 5 |
| Arylsulfatase B; isoform CRA_a |
| Protein O-GlcNAcase (OGA) (EC 3.2.1.169) (Beta-N-acetylglucosaminidase) (Beta-N-acetylhexosaminidase) (Beta-hexosaminidase) (Meningioma-expressed antigen 5) (N-acetyl-beta-D-glucosaminidase) (N-acetyl-beta-glucosaminidase) (Nuclear cytoplasmic O-GlcNAcase and acetyltransferase) (NCOAT) |
| Multifunctional fusion protein [Includes: L-glutamate gamma-semialdehyde dehydrogenase (EC 1.2.1.88) (L-glutamate gamma-semialdehyde dehydrogenase); Delta-1-pyrroline-5-carboxylate dehydrogenase (P5C dehydrogenase)] |
| cDNA FLJ54090; highly similar to 4F2 cell-surface antigen heavy chain |
| Ezrin |
| HEXA protein (Fragment) |
| Arginine/serine-rich splicing factor 6 variant (Fragment) |
| Replication protein A3; 14kDa (Replication protein A3; 14kDa; isoform CRA_b) (cDNA; FLJ92105; Homo sapiens replication protein A3; 14kDa (RPA3); mRNA) |
| 28S ribosomal protein S31; mitochondrial (MRP-S31) (S31mt) (Imogen 38) (Mitochondrial small ribosomal subunit protein mS31) |
| Malic enzyme |
| Uncharacterized protein DKFZp686M0619 (Fragment) |
| DNA topoisomerase 2-alpha (EC 5.6.2.2) (DNA topoisomerase II; alpha isozyme) |
| Dehydrogenase/reductase SDR family member 4 (EC 1.1.1.184) (NADPH-dependent carbonyl reductase/NADP-retinol dehydrogenase) (CR) (PHCR) (NADPH-dependent retinol dehydrogenase/reductase) (NRDR) (humNRDR) (Peroxisomal short-chain alcohol dehydrogenase) (PSCD) (SCAD-SRL) (Short chain dehydrogenase/reductase family 25C member 2) (Short-chain dehydrogenase/reductase family member 4) |
| Ribosome biogenesis protein BOP1 (Block of proliferation 1 protein) |
| Inositol-1-monophosphatase (EC 3.1.3.25) |
| Stathmin (Fragment) |
| Monofunctional C1-tetrahydrofolate synthase; mitochondrial |
| Nesprin-1 (Enaptin) (KASH domain-containing protein 1) (KASH1) (Myocyte nuclear envelope protein 1) (Myne-1) (Nuclear envelope spectrin repeat protein 1) (Synaptic nuclear envelope protein 1) (Syne-1) |
| Dihydrofolate reductase (EC 1.5.1.3) (cDNA; FLJ93028; Homo sapiens dihydrofolate reductase (DHFR); mRNA) |
| Trafficking protein particle complex subunit |
| DNA helicase (EC 3.6.4.12) |
| NADPH:adrenodoxin oxidoreductase; mitochondrial (EC 1.18.1.6) |
| N-alpha-acetyltransferase 15; NatA auxiliary subunit |
| Quinone oxidoreductase (EC 1.6.5.5) (NADPH:quinone reductase) (Zeta-crystallin) |

cDNA FLJ75877; highly similar to Homo sapiens 5'-nucleotidase; cytosolic II (NT5C2); mRNA

cDNA FLJ54257; highly similar to Leiomodin-1

DNA-directed RNA polymerase subunit beta (EC 2.7.7.6)

DNA helicase (EC 3.6.4.12) (Fragment)

Membrane-associated progesterone receptor component 2 (Progesterone membrane-binding protein) (Steroid receptor protein DG6)

Nestin; isoform CRA_c

Dipeptidylpeptidase III isoform 1 variant (Fragment)

Protein SET

Sister chromatid cohesion protein PDS5 homolog B (Androgen-induced proliferation inhibitor) (Androgen-induced prostate proliferative shutoff-associated protein AS3)

Plectin (PCN) (PLTN) (Hemidesmosomal protein 1) (HD1) (Plectin-1)

Serine threonine kinase 39 isoform B (Fragment)

Family with sequence similarity 62 (C2 domain containing); member A; isoform CRA_a

Protein NipSnap homolog 3A (NipSnap3A) (Protein NipSnap homolog 4) (NipSnap4) (Target for Salmonella secreted protein C) (TassC)

NUMA1 variant protein (Fragment)

Myoferlin (Fer-1-like protein 3)

cDNA FLJ53963; highly similar to Leukocyte elastase inhibitor

Splicing factor 3B subunit 3 (Pre-mRNA-splicing factor SF3b 130 kDa subunit) (SF3b130) (STAF130) (Spliceosome-associated protein 130) (SAP 130)

Spectrin beta chain

DNA replication licensing factor MCM6 (EC 3.6.4.12) (p105MCM)

Lamin-B1

Collagen alpha-1(XII) chain

EH-domain containing 2; isoform CRA_a

Unconventional myosin-Ib

Casein kinase II subunit alpha

U5 small nuclear ribonucleoprotein 200 kDa helicase (EC 3.6.4.13) (Activating signal cointegrator 1 complex subunit 3-like 1) (BRR2 homolog) (U5 snRNP-specific 200 kDa protein) (U5-200KD)

HCG1991735; isoform CRA_a

Band 4.1-like protein 2 (Generally expressed protein 4.1) (4.1G)

Unconventional myosin-Ic

Sulfatase modifying factor 2 isoform 2

**vii. 84 elements included exclusively in "100 µM c-di-AMP+*Pg* LPS↓":**

Phosphoserine phosphatase (Fragment)

Putative 60S ribosomal protein L39-like 5 (60S ribosomal protein L39 pseudogene 5)

Ribosomal protein L38; isoform CRA_a

SH2 domain-containing protein 4A (Protein SH(2)A) (Protein phosphatase 1 regulatory subunit 38)

| |
|---|
| cDNA FLJ76855; highly similar to Homo sapiens exportin 7 (XPO7); mRNA |
| Ubiquitin-fold modifier-conjugating enzyme 1 (Ufm1-conjugating enzyme 1) |
| 40S ribosomal protein S5 (Fragment) |
| Coatomer subunit gamma |
| Protein S100-A6 (Calcyclin) (Growth factor-inducible protein 2A9) (MLN 4) (Prolactin receptor-associated protein) (PRA) (S100 calcium-binding protein A6) |
| DCC-interacting protein 13-beta (Dip13-beta) (Adapter protein containing PH domain; PTB domain and leucine zipper motif 2) |
| 60S ribosomal protein L18a |
| Phosphoribosyl pyrophosphate synthetase-associated protein 1; isoform CRA_a |
| Dr1-associated corepressor |
| Ribonucloprotein |
| Proteasome subunit beta type-5 (EC 3.4.25.1) (Macropain epsilon chain) (Multicatalytic endopeptidase complex epsilon chain) (Proteasome chain 6) (Proteasome epsilon chain) (Proteasome subunit MB1) (Proteasome subunit X) |
| Farnesyl pyrophosphate synthase (FPP synthase) (FPS) (EC 2.5.1.10) ((2E;6E)-farnesyl diphosphate synthase) (Dimethylallyltranstransferase) (EC 2.5.1.1) (Farnesyl diphosphate synthase) (Geranyltranstransferase) |
| cDNA FLJ76072; highly similar to Homo sapiens GIPC PDZ domain containing family; member 1 (GIPC1); transcript variant 1; mRNA |
| Phosphoserine aminotransferase (EC 2.6.1.52) |
| Pyruvate kinase (EC 2.7.1.40) |
| Tyrosine--tRNA ligase (EC 6.1.1.1) (Tyrosyl-tRNA synthetase) (Fragment) |
| Ribosomal protein L7; isoform CRA_a |
| Ras-related protein R-Ras2 (Ras-like protein TC21) (Teratocarcinoma oncogene) |
| Protein bicaudal D homolog 2 (Bic-D 2) |
| Squamous cell carcinoma antigen recognized by T-cells 3 |
| Basic leucine zipper and W2 domains 1, isoform CRA_a |
| 40S ribosomal protein S15 |
| V-type proton ATPase subunit C |
| Rho-associated protein kinase (EC 2.7.11.1) |
| Alanine--tRNA ligase; cytoplasmic (EC 6.1.1.7) (Alanyl-tRNA synthetase) (AlaRS) (Renal carcinoma antigen NY-REN-42) |
| cDNA FLJ54534; highly similar to Homo sapiens cysteinyl-tRNA synthetase (CARS); transcript variant 3; mRNA |
| Signal recognition particle subunit SRP68 (SRP68) (Signal recognition particle 68 kDa protein) |
| Eukaryotic translation initiation factor 3 subunit C (eIF3c) (Eukaryotic translation initiation factor 3 subunit 8) (eIF3 p110) |
| Heat shock protein 90kDa alpha (Cytosolic); class B member 1; isoform CRA_a |
| cDNA FLJ12454 fis; clone NT2RM1000555; highly similar to UNR PROTEIN (cDNA FLJ12466 fis; clone NT2RM1000826; highly similar to UNR PROTEIN) |
| Actinin alpha 4 isoform 1 (Fragment) |
| Fascin |
| High density lipoprotein binding protein (Vigilin); isoform CRA_a (Vigilin) |

| |
|---|
| Programmed cell death 6-interacting protein (PDCD6-interacting protein) (ALG-2-interacting protein 1) (ALG-2-interacting protein X) (Hp95) |
| Rab GDP dissociation inhibitor |
| Signal recognition particle subunit SRP72 |
| Glutamine--fructose-6-phosphate aminotransferase [isomerizing] 1 (EC 2.6.1.16) (D-fructose-6-phosphate amidotransferase 1) (Glutamine:fructose-6-phosphate amidotransferase 1) (GFAT 1) (GFAT1) (Hexosephosphate aminotransferase 1) |
| L-lactate dehydrogenase (EC 1.1.1.27) |
| Proteasome subunit beta (EC 3.4.25.1) (Fragment) |
| 40S ribosomal protein S3 (EC 4.2.99.18) (Small ribosomal subunit protein uS3) |
| Aspartate aminotransferase (EC 2.6.1.1) |
| Glucose-6-phosphate 1-dehydrogenase (EC 1.1.1.49) |
| Glycyl-tRNA synthetase |
| Thrombospondin-1 (Glycoprotein G) |
| Serine/threonine-protein phosphatase 2A 56 kDa regulatory subunit |
| DDX39B (HCG2005638; isoform CRA_a) |
| Eukaryotic translation initiation factor 3 subunit L (eIF3l) (Eukaryotic translation initiation factor 3 subunit 6-interacting protein) (Eukaryotic translation initiation factor 3 subunit E-interacting protein) |
| Nuclear autoantigenic sperm protein (NASP) |
| ATP-citrate synthase (EC 2.3.3.8) (ATP-citrate (pro-S-)-lyase) (Citrate cleavage enzyme) |
| Phosphatidylinositol transfer protein beta isoform (PI-TP-beta) (PtdIns transfer protein beta) (PtdInsTP beta) |
| Vacuolar protein sorting-associated protein 35 (hVPS35) (Maternal-embryonic 3) (Vesicle protein sorting 35) |
| Eukaryotic translation initiation factor 5A-1 (eIF-5A-1) (eIF-5A1) (Eukaryotic initiation factor 5A isoform 1) (eIF-5A) (Rev-binding factor) (eIF-4D) |
| Clathrin heavy chain |
| 60S ribosomal protein L3 (HIV-1 TAR RNA-binding protein B) (TARBP-B) (Large ribosomal subunit protein uL3) |
| N-acylaminoacyl-peptide hydrolase, isoform CRA_b |
| Proliferation-associated 2G4; 38kDa; isoform CRA_a |
| Heterogeneous nuclear ribonucleoprotein Q (hnRNP Q) (Glycine- and tyrosine-rich RNA-binding protein) (GRY-RBP) (NS1-associated protein 1) (Synaptotagmin-binding; cytoplasmic RNA-interacting protein) |
| Glucan; branching enzyme 1 variant (Fragment) |
| 26S proteasome non-ATPase regulatory subunit 1 |
| Testicular tissue protein Li 192 |
| Mitotic checkpoint protein BUB3 (Fragment) |
| Eukaryotic translation initiation factor 3 subunit A (eIF3a) (Eukaryotic translation initiation factor 3 subunit 10) (eIF-3-theta) |
| Pumilio homolog 1 (Fragment) |
| Bifunctional glutamate/proline--tRNA ligase (Bifunctional aminoacyl-tRNA synthetase) (Cell proliferation-inducing gene 32 protein) (Glutamatyl-prolyl-tRNA |

| |
| --- |
| synthetase) [Includes: Glutamate--tRNA ligase (EC 6.1.1.17) (Glutamyl-tRNA synthetase) (GluRS); Proline--tRNA ligase (EC 6.1.1.15) (Prolyl-tRNA synthetase)] |
| Tubulin alpha-1B chain (Alpha-tubulin ubiquitous) (Tubulin K-alpha-1) (Tubulin alpha-ubiquitous chain) [Cleaved into: Detyrosinated tubulin alpha-1B chain] |
| Testicular secretory protein Li 63 (Ubiquitin-activating enzyme E1 (A1S9T and BN75 temperature sensitivity complementing); isoform CRA_a) |
| N-alpha-acetyltransferase 10 (EC 2.3.1.255) (N-terminal acetyltransferase complex ARD1 subunit homolog A) (hARD1) (NatA catalytic subunit Naa10) |
| Catenin (Cadherin-associated protein); alpha 1; 102kDa; isoform CRA_b (Epididymis secretory sperm binding protein) |
| Asparagine--tRNA ligase; cytoplasmic (EC 6.1.1.22) (Asparaginyl-tRNA synthetase) (AsnRS) (Asparaginyl-tRNA synthetase 1) |
| Spermidine synthase (SPDSY) (EC 2.5.1.16) (Putrescine aminopropyltransferase) |
| Kinesin-like protein |
| Epididymis luminal protein 33 (Epididymis secretory sperm binding protein) (Epididymis secretory sperm binding protein Li 72p) (Heat shock 70kDa protein 8; isoform CRA_a) |
| ARP3 actin-related protein 3 homolog (Yeast); isoform CRA_a |
| Ribosome assembly factor mrt4 |
| DNA damage-binding protein 1 (DDB p127 subunit) (DNA damage-binding protein a) (DDBa) (Damage-specific DNA-binding protein 1) (HBV X-associated protein 1) (XAP-1) (UV-damaged DNA-binding factor) (UV-damaged DNA-binding protein 1) (UV-DDB 1) (XPE-binding factor) (XPE-BF) (Xeroderma pigmentosum group E-complementing protein) (XPCe) |
| Testis secretory sperm-binding protein Li 197a |
| SEC31-like 1 (S. cerevisiae), isoform CRA_d |
| Seryl-tRNA synthetase variant (Fragment) |
| Vacuolar protein sorting-associated protein 26A (Vesicle protein sorting 26A) (hVPS26) |
| Epididymis luminal protein 70 (Moesin; isoform CRA_a) |
