# Supplementary material for: Activation of cellular responses by cyclic dinucleotides and porphyromonas gingivalis lipopolysaccharide: a proteomic study on gingival fibroblasts
Source: J Oral Microbiol. 2024 Dec 9;17(1):2431453. doi: 10.1080/20002297.2024.2431453 (PMC11632945; doi:10.1080/20002297.2024.2431453)
Supplement: Table_S2_up_regulated_proteins.pdf [file ZJOM_A_2431453_SM5360.pdf]

Table S2. List of upregulated proteins with a measurable fold change (Log2 fold change ≥ 0.5 and $p \leq 0.05$) in different treatment groups (100 µM c-di-AMP+*Pg* LPS or c-di-GMP+*Pg* LPS or *Pg* LPS)

S/No	***Treatment/Venn Diagram group***
i.	**12 common elements in "100 µM c-di-AMP+*Pg* LPS↑↑", "100 µM c-di-GMP+*Pg* LPS↑↑" and "*Pg* LPS↑↑":**
	Transmembrane protein 263
	Superoxide dismutase (EC 1.15.1.1) (Fragment)
	Cell proliferation-inducing protein 41
	Slingshot homolog 3 (Drosophila); isoform CRA_f
	Epididymis secretory sperm binding protein
	ATP synthase subunit e; mitochondrial (ATPase subunit e) (ATP synthase membrane subunit e) [Cleaved into: ATP synthase subunit e; mitochondrial; N-terminally processed]
	Ferritin
	Glucosamine-6-phosphate isomerase (EC 3.5.99.6) (Glucosamine-6-phosphate deaminase)
	ADP-sugar pyrophosphatase
	SH3 and PX domain-containing protein 2B (Adapter protein HOFI) (Factor for adipocyte differentiation 49) (Tyrosine kinase substrate with four SH3 domains)
	Ubiquitin/ISG15-conjugating enzyme E2 L6 (EC 2.3.2.23) (E2 ubiquitin-conjugating enzyme L6) (Retinoic acid-induced gene B protein) (RIG-B) (UbcH8) (Ubiquitin carrier protein L6) (Ubiquitin-protein ligase L6)
	Alpha-endosulfine
ii.	**10 common elements in "100 µM c-di-GMP+*Pg* LPS↑↑" and "*Pg* LPS↑↑":**
	cDNA FLJ59612; highly similar to Lactadherin
	Phospholipase D family; member 3; isoform CRA_b
	Aldo-keto reductase family 1 member C3
	Ubiquitin-like modifier-activating enzyme 7 (Ubiquitin-activating enzyme 7) (D8) (Ubiquitin-activating enzyme E1 homolog)
	Triosephosphate isomerase (EC 5.3.1.1)
	Prolyl endopeptidase FAP
	Elongation factor 1-alpha
	Protein kinase cAMP-dependent regulatory type II alpha isoform 1 (Fragment)
	Histone deacetylase complex subunit SAP18
	Fibulin-1
iii.	**8 common elements in "100 µM c-di-AMP+*Pg* LPS↑↑" and "*Pg* LPS↑↑":**
	Isocitrate dehydrogenase [NAD] subunit gamma; mitochondrial (Isocitric dehydrogenase subunit gamma) (NAD(+)-specific ICDH subunit gamma)
	Protein kinase C inhibitor-2
	NADH dehydrogenase [ubiquinone] iron-sulfur protein 8; mitochondrial (Fragment)

	cDNA FLJ51688; highly similar to Cleavage stimulation factor 50 kDa subunit
	Mago nashi protein (Mago-nashi homolog; isoform CRA_c)
	Putative phospholipase B-like 2 (EC 3.1.1.-) (76 kDa protein) (p76) (LAMA-like protein 2) (Lamina ancestor homolog 2) (Phospholipase B domain-containing protein 2) [Cleaved into: Putative phospholipase B-like 2 32 kDa form; Putative phospholipase B-like 2 45 kDa form]
	cDNA FLJ53327; highly similar to Gelsolin
	Acyl carrier protein; mitochondrial (Fragment)
iv.	**38 elements included exclusively in "*Pg* LPS↑↑":**
	Calpain small subunit 1 (CSS1) (Calcium-activated neutral proteinase small subunit) (CANP small subunit) (Calcium-dependent protease small subunit) (CDPS) (Calcium-dependent protease small subunit 1) (Calpain regulatory subunit)
	cDNA; FLJ79464; highly similar to Homo sapiens aquarius (Fragment)
	HCG26477 (Ribosomal protein S28; isoform CRA_a) (cDNA; FLJ92192; Homo sapiens ribosomal protein S28 (RPS28); mRNA)
	ATPase family AAA domain-containing protein 3A (Fragment)
	Cullin-1
	Epididymis secretory protein Li 108 (Tropomyosin 4; isoform CRA_a)
	NADH dehydrogenase (Ubiquinone) 1 alpha subcomplex; 4; 9kDa; isoform CRA_b
	NF-kappa-B essential modulator
	Methionine-R-sulfoxide reductase B3 (MsrB3) (EC 1.8.4.12) (EC 1.8.4.14)
	Peroxisomal carnitine O-octanoyltransferase
	Carboxymethylenebutenolidase homolog (EC 3.1.-.-)
	GDP-mannose pyrophosphorylase B, isoform CRA_b OS=Homo sapiens OX=9606 GN=GMPPB PE=4 SV=1;tr|A0A024R329|A0A024R329_HUMAN GDP-mannose pyrophosphorylase B, isoform CRA_a
	Protein S100-A10 (Calpactin I light chain) (Calpactin-1 light chain) (Cellular ligand of annexin II) (S100 calcium-binding protein A10) (p10 protein) (p11)
	Protein S100-A4 (Calvasculin) (Metastasin) (Placental calcium-binding protein) (Protein Mts1) (S100 calcium-binding protein A4)
	Tissue factor (Fragment)
	ATP synthase subunit gamma
	Calpain-1 catalytic subunit (EC 3.4.22.52) (Calcium-activated neutral proteinase 1) (CANP 1) (Calpain mu-type) (Calpain-1 large subunit) (Cell proliferation-inducing gene 30 protein) (Micromolar-calpain) (muCANP)
	Epididymis luminal protein 113 (Epididymis secretory sperm binding protein) (Vimentin isoform 1)
	Rho GTPase-activating protein 17 (Rho-type GTPase-activating protein 17) (RhoGAP interacting with CIP4 homologs protein 1) (RICH-1)
	Glutathione S-transferase (EC 2.5.1.18)
	Shwachman-Bodian-Diamond syndrome isoform 1 (Fragment)
	Protein tyrosine phosphatase non-receptor type 11
	cDNA FLJ52710; highly similar to Abhydrolase domain-containing protein 14B
	Beta-hexosaminidase (EC 3.2.1.52)
	EBNA1 binding protein 2 (EBNA1 binding protein 2 variant) (EBNA1 binding protein 2; isoform CRA_a) (EBNA1BP2 protein)

	Serpin B6 (Serpin peptidase inhibitor; clade B (Ovalbumin); member 6; isoform CRA_d)
	Myotrophin (Myotrophin; isoform CRA_a) (V-1 protein) (cDNA FLJ31663 fis; clone NT2RI2004504; highly similar to MYOTROPHIN)
	cDNA FLJ38393 fis; clone FEBRA2007212
	V-crk sarcoma virus CT10 oncogene homolog (Avian)
	Liprin-beta-1 (Protein tyrosine phosphatase receptor type f polypeptide-interacting protein-binding protein 1) (PTPRF-interacting protein-binding protein 1) (hSGT2)
	Vesicle-fusing ATPase
	ATP-dependent RNA helicase DDX1 (DEAD (Asp-Glu-Ala-Asp) box polypeptide 1; isoform CRA_d) (DEAD box polypeptide 1) (cDNA; FLJ94573; Homo sapiens DEAD (Asp-Glu-Ala-Asp) box polypeptide 1 (DDX1); mRNA)
	UMP-CMP kinase (EC 2.7.4.14) (Deoxycytidylate kinase) (CK) (dCMP kinase) (Nucleoside-diphosphate kinase) (EC 2.7.4.6) (Uridine monophosphate/cytidine monophosphate kinase) (UMP/CMP kinase) (UMP/CMPK)
	Vacuolar protein sorting-associated protein 26A (Vesicle protein sorting 26A) (hVPS26)Cytochrome c oxidase subunit 6A; mitochondrial (Cytochrome c oxidase polypeptide VIa)
	H.sapiens ras-related Hrab4 protein
	Leucine-rich repeat protein SHOC-2 (Protein soc-2 homolog) (Protein sur-8 homolog)
	Telomeric repeat-binding factor 2-interacting protein 1 (TERF2-interacting telomeric protein 1) (TRF2-interacting telomeric protein 1) (Dopamine receptor-interacting protein 5) (Repressor/activator protein 1 homolog) (RAP1 homolog) (hRap1)
	cDNA FLJ60960; highly similar to Phosphatidylinositol 4-kinase alpha
v.	**53 common elements in "100 μM c-di-AMP+*Pg* LPS↑↑" and "100 μM c-di-GMP+*Pg* LPS↑↑":**
	Ubiquitin-like protein ISG15 (Interferon-induced 15 kDa protein) (Interferon-induced 17 kDa protein) (IP17) (Ubiquitin cross-reactive protein) (hUCRP)
	Deoxynucleoside triphosphate triphosphohydrolase SAMHD1 (dNTPase) (EC 3.1.5.-) (Dendritic cell-derived IFNG-induced protein) (DCIP) (Monocyte protein 5) (MOP-5) (SAM domain and HD domain-containing protein 1) (hSAMHD1)
	cDNA FLJ78682; highly similar to Homo sapiens 2'-5'-oligoadenylate synthetase 3; 100kDa (OAS3); mRNA
	Plasminogen activator inhibitor 2 (PAI-2) (Monocyte Arg-serpin) (Placental plasminogen activator inhibitor) (Serpin B2) (Urokinase inhibitor)
	Signal transducer and activator of transcription
	cDNA FLJ53509; highly similar to Galectin-3-binding protein
	Polyribonucleotide nucleotidyltransferase 1; mitochondrial (EC 2.7.7.8) (3'-5' RNA exonuclease OLD35) (PNPase old-35) (Polynucleotide phosphorylase 1) (PNPase 1) (Polynucleotide phosphorylase-like protein)
	TAP1
	Apolipoprotein L; 2; isoform CRA_a
	Signal transducer and activator of transcription 1-alpha/beta (Transcription factor ISGF-3 components p91/p84)

Nuclear autoantigen Sp-100 (Fragment)
eIF2AK2 protein
Protein PML (Promyelocytic leukemia protein) (RING finger protein 71) (Tripartite motif-containing protein 19)
MHC class I antigen
Epididymis secretory protein Li 106
Beta-2-microglobulin
SAR1 gene homolog A (S. cerevisiae); isoform CRA_a (Small GTP-binding protein)
Thymidine phosphorylase (TP) (EC 2.4.2.4) (TdRPase)
TAPBP
Interferon; gamma-inducible protein 16; isoform CRA_a (Interferon; gamma-inducible protein 16; isoform CRA_b)
E3 ubiquitin/ISG15 ligase TRIM25 (EC 6.3.2.n3) (Estrogen-responsive finger protein) (RING finger protein 147) (RING-type E3 ubiquitin transferase) (EC 2.3.2.27) (RING-type E3 ubiquitin transferase TRIM25) (Tripartite motif-containing protein 25) (Ubiquitin/ISG15-conjugating enzyme TRIM25) (Zinc finger protein 147)
Proteasome subunit beta (EC 3.4.25.1)
ADP-ribosylation factor-like protein 1
Adenosine deaminase; RNA-specific isoform ADAR-a variant (Fragment)
Glycerol kinase (GK) (Glycerokinase) (EC 2.7.1.30) (ATP:glycerol 3-phosphotransferase)
Torsin-1A-interacting protein 1
HCG2001986; isoform CRA_a
Cytochrome b-5 isoform 1 variant (Fragment)
MHC class I antigen (Fragment)
Electron-transfer-flavoprotein alpha polypeptide isoform 1 (Epididymis secretory sperm binding protein) (Fragment)
Cytochrome b-c1 complex subunit 2; mitochondrial (Complex III subunit 2) (Core protein II) (Ubiquinol-cytochrome-c reductase complex core protein 2)
Peroxidasin homolog (EC 1.11.1.7) (Melanoma-associated antigen MG50) (Vascular peroxidase 1) (p53-responsive gene 2 protein)
Matrix metalloproteinase 14 (Membrane-inserted)
PGRMC1 protein (Progesterone receptor membrane component 1) (Progesterone receptor membrane component 1; isoform CRA_a) (cDNA; FLJ94602; Homo sapiens progesterone receptor membrane component 1 (PGRMC1);mRNA)
cDNA FLJ54595; highly similar to Golgi reassembly-stacking protein 2
Glucosylceramidase (EC 3.2.1.45)
ERO1-like protein alpha (ERO1-L) (ERO1-L-alpha) (EC 1.8.4.-) (Endoplasmic oxidoreductin-1-like protein) (Endoplasmic reticulum oxidoreductase alpha) (Oxidoreductin-1-L-alpha)
Integrin alpha-2 (CD49 antigen-like family member B) (Collagen receptor) (Platelet membrane glycoprotein Ia) (GPIa) (VLA-2 subunit alpha) (CD antigen CD49b)
Leupaxin
Mitochondrial aldehyde dehydrogenase 2 variant (Fragment)
Aminopeptidase (EC 3.4.11.-)

	Interferon regulatory factor 9 (IRF-9) (IFN-alpha-responsive transcription factor subunit) (ISGF3 p48 subunit) (Interferon-stimulated gene factor 3 gamma) (ISGF-3 gamma) (Transcriptional regulator ISGF3 subunit gamma)
	cDNA FLJ75441; highly similar to Homo sapiens vaccinia related kinase 1 (VRK1); mRNA
	Nitric oxide synthase-interacting protein
	Interferon-induced GTP-binding protein Mx1 (Interferon-induced protein p78) (IFI-78K) (Interferon-regulated resistance GTP-binding protein MxA) (Myxoma resistance protein 1) (Myxovirus resistance protein 1) [Cleaved into: Interferon-induced GTP-binding protein Mx1; N-terminally processed]
	Interferon-induced protein with tetratricopeptide repeats 3; isoform CRA_a (Interferon-induced protein with tetratricopeptide repeats 3; isoform CRA_b) (cDNA FLJ75638; highly similar to Homo sapiens interferon-induced protein with tetratricopeptide repeats 3 (IFIT3); mRNA)
	Interferon-induced protein with tetratricopeptide repeats 1 (IFIT-1) (Interferon-induced 56 kDa protein) (IFI-56K) (P56)
	Interferon-induced GTP-binding protein Mx2 (Interferon-regulated resistance GTP-binding protein MxB) (Myxovirus resistance protein 2) (p78-related protein)
	cDNA FLJ75060; highly similar to Homo sapiens 2'-5'-oligoadenylate synthetase 2; 69/71kDa; mRNA
	cDNA FLJ44001 fis; clone TESTI4022716; highly similar to Probable ATP-dependent RNA helicase DDX58
	Filamin A-interacting protein 1-like (130 kDa GPBP-interacting protein) (90 kDa GPBP-interacting protein) (Protein down-regulated in ovarian cancer 1) (DOC-1)
	E3 ubiquitin-protein ligase DTX3L (EC 2.3.2.27) (B-lymphoma- and BAL-associated protein) (Protein deltex-3-like) (RING-type E3 ubiquitin transferase DTX3L) (Rhysin-2) (Rhysin2)
	POLDIP3 protein (Fragment)
vi.	**101 elements included exclusively in "100 µM c-di-GMP+ *Pg* LPS↑↑":**
	Matrix metalloproteinase 1 preproprotein variant (Fragment)
	Cell migration-inducing and hyaluronan-binding protein (EC 3.2.1.35)
	cDNA FLJ34004 fis; clone FCBBF1000232; highly similar to Cytochrome P450 51A1
	Telomerase RNA component interacting RNase (EC 3.1.13.-) (Exoribonuclease TRIR)
	Serpin peptidase inhibitor; clade E (Nexin; plasminogen activator inhibitor type 1); member 1; isoform CRA_b
	Aldo-keto reductase family 1; member B1 (Aldose reductase); isoform CRA_a
	Nuclear factor NF-kappa-B p100 subunit
	Syndecan binding protein (Syntenin); isoform CRA_c
	cDNA FLJ16785 fis; clone NT2RI2015342; highly similar to Solute carrier family 2; facilitated glucose transporter member 1
	Thrombospondin-2
	Rho family GTPase 3; isoform CRA_b
	Sequestosome-1 (EBI3-associated protein of 60 kDa) (EBIAP) (p60) (Phosphotyrosine-independent ligand for the Lck SH2 domain of 62 kDa) (Ubiquitin-binding protein p62)
	Insulin-like growth factor binding protein 5, isoform CRA_a

ENO2 protein (Enolase 2 (Gamma; neuronal); isoform CRA_a) (Epididymis secretory protein Li 279) (cDNA FLJ42665 fis; clone BRAMY2019989; highly similar to Gamma-enolase) (cDNA; FLJ92734; Homo sapiens enolase 2; (gamma; neuronal) (ENO2); mRNA)
Long-chain-fatty-acid--CoA ligase 4 (EC 6.2.1.3) (Arachidonate--CoA ligase) (EC 6.2.1.15) (Long-chain acyl-CoA synthetase 4) (LACS 4)
Tumor necrosis factor receptor superfamily member 11B (Osteoclastogenesis inhibitory factor) (Osteoprotegerin)
cDNA; FLJ94074; highly similar to Homo sapiens caspase 1; apoptosis-related cysteine protease(interleukin 1; beta; convertase) (CASP1); transcript variantalpha; mRNA
Pyridoxal phosphate homeostasis protein (PLP homeostasis protein) (Proline synthase co-transcribed bacterial homolog protein)
Isopentenyl-diphosphate Delta-isomerase 1 (EC 5.3.3.2) (Isopentenyl pyrophosphate isomerase 1) (IPP isomerase 1) (IPPI1)
Thrombospondin-1 (Glycoprotein G)
Full-length cDNA clone CS0DJ015YJ12 of T cells (Jurkat cell line) of Homo sapiens (human) (PSME2 protein)
Proteasome subunit beta (EC 3.4.25.1) (Fragment)
Epididymis secretory sperm binding protein (Macrophage migration inhibitory factor) (Fragment)
Uncharacterized protein DKFZp686M0430
cDNA FLJ58182; highly similar to Protein CYR61
cDNA FLJ75881; highly similar to Homo sapiens transferrin receptor (p90; CD71) (TFRC); mRNA
Guanylate-binding protein 1 (EC 3.6.5.-) (GTP-binding protein 1) (GBP-1) (HuGBP-1) (Guanine nucleotide-binding protein 1) (Interferon-induced guanylate-binding protein 1)
EGF-like repeat and discoidin I-like domain-containing protein 3 (Developmentally-regulated endothelial cell locus 1 protein) (Integrin-binding protein DEL1)
cDNA FLJ57805; highly similar to Homo sapiens paraspeckle component 1 (PSPC1); transcript variant alpha; mRNA
Fibroblast growth factor (FGF)
cDNA FLJ59425; highly similar to SH3-domain kinase-binding protein 1
DNA mismatch repair protein Msh6 (hMSH6) (G/T mismatch-binding protein) (GTBP) (GTMBP) (MutS protein homolog 6) (MutS-alpha 160 kDa subunit) (p160)
Transmembrane protein 14C variant (Fragment)
Phospholipase A-2-activating protein (PLA2P) (PLAP)
Fibronectin type III domain-containing protein 3B (Factor for adipocyte differentiation 104) (HCV NS5A-binding protein 37)
Cdc42-interacting protein 4 (Thyroid hormone receptor interactor 10; isoform CRA_a)
MAP2K3 protein (Mitogen-activated protein kinase kinase 3 isoform B) (cDNA; FLJ96569; Homo sapiens mitogen-activated protein kinase kinase 3 (MAP2K3);transcript variant B; mRNA)
Procollagen-lysine;2-oxoglutarate 5-dioxygenase 2

Glycogen [starch] synthase (EC 2.4.1.11)
Epididymis luminal protein 60 (Epididymis secretory sperm binding protein)
Nicotinamide phosphoribosyltransferase (NAmPRTase) (EC 2.4.2.12)
Synaptosomal-associated protein 29 (SNAP-29) (Soluble 29 kDa NSF attachment protein) (Vesicle-membrane fusion protein SNAP-29)
Degenerative spermatocyte homolog 1; lipid desaturase (Drosophila); isoform CRA_a
Prolyl 4-hydroxylase subunit alpha-1 (4-PH alpha-1) (EC 1.14.11.2) (Procollagen-proline;2-oxoglutarate-4-dioxygenase subunit alpha-1)
TMEM214 protein (Fragment)
Laminin; gamma 1 (Formerly LAMB2); isoform CRA_a
Kinesin light chain 2 (KLC 2)
Ectopic P granules protein 5 homolog
Sorting nexin-3 (Protein SDP3)
Phosphoglycerate mutase (EC 5.4.2.11) (EC 5.4.2.4) (Fragment)
RAB7; member RAS oncogene family; isoform CRA_a (RAB7A; member RAS oncogene family)
cDNA; FLJ96901; highly similar to Homo sapiens Rac GTPase activating protein 1 (RACGAP1); mRNA
Sorting nexin 1 isoform a variant (Fragment)
cDNA FLJ35762 fis; clone TESTI2004793; moderately similar to Homo sapiens NY-REN-2 antigen mRNA
Integrin; alpha 5 (Fibronectin receptor; alpha polypeptide); isoform CRA_b
p4HA2 protein
Hypoxia up-regulated protein 1
Oxysterol-binding protein 1
Endothelin-converting enzyme 1 (cDNA FLJ59212; highly similar to Endothelin-converting enzyme 1)
ATP-dependent 6-phosphofructokinase; platelet type (ATP-PFK) (PFK-P) (EC 2.7.1.11) (6-phosphofructokinase type C) (Phosphofructo-1-kinase isozyme C) (PFK-C) (Phosphohexokinase)
Phosphoinositide phospholipase C (EC 3.1.4.11) (Fragment)
Stomatin; isoform CRA_a
Probable ATP-dependent RNA helicase DDX17
Fibronectin 1; isoform CRA_n
Procollagen-lysine 1, 2-oxoglutarate 5-dioxygenase 1, isoform CRA_a
Epididymis secretory sperm binding protein Li 89n
Chromosome 12 open reading frame 10 (Chromosome 12 open reading frame 10; isoform CRA_b)
Transportin 1; isoform CRA_a
cDNA FLJ44469 fis; clone UTERU2026090; highly similar to Cartilage-associated protein
Elongin-C (Fragment)
Putative hydroxypyruvate isomerase (Fragment)

	cDNA FLJ77398; highly similar to Homo sapiens UDP-glucose ceramide glucosyltransferase-like 1; transcript variant 2; mRNA
	Zinc transporter ZIP14 (Fragment)
	Coiled-coil-helix-coiled-coil-helix domain-containing protein 2 (Aging-associated gene 10 protein) (HCV NS2 trans-regulated protein) (NS2TP)
	Truncated Niemann-Pick C1
	Chromosome 15 open reading frame 48; isoform CRA_a
	Protein tweety homolog
	Amyloid-beta precursor protein
	3-hydroxy-3-methylglutaryl coenzyme A synthase (HMG-CoA synthase) (EC 2.3.3.10)
	Low-density lipoprotein receptor-related protein 10 (LRP-10)
	Protein mono-ADP-ribosyltransferase PARP14 (EC 2.4.2.-) (ADP-ribosyltransferase diphtheria toxin-like 8) (ARTD8) (B aggressive lymphoma protein 2) (Poly [ADP-ribose] polymerase 14) (PARP-14)
	Vacuolar protein sorting 28-like protein isoform 2 (Fragment)
	Collagen triple helix repeat-containing protein 1 (Protein NMTC1)
	Methyl-CpG-binding protein 2 (MeCp-2 protein) (MeCp2)
	Coiled-coil domain-containing protein 86 (Cytokine-induced protein with coiled-coil domain)
	cDNA FLJ77229; highly similar to Homo sapiens GRIP1 associated protein 1 (GRIPAP1); transcript variant 1; mRNA
	Urokinase plasminogen activator preproprotein variant (Fragment)
	Receptor-interacting serine/threonine-protein kinase 2 (EC 2.7.11.1) (Tyrosine-protein kinase RIPK2)
	Membrane metallo-endopeptidase
	Calcium-transporting ATPase type 2C member 1 (Fragment)
	Carnitine O-palmitoyltransferase 2; mitochondrial
	Inactive tyrosine-protein kinase PEAK1 (Pseudopodium-enriched atypical kinase 1) (Sugen kinase 269) (Tyrosine-protein kinase SgK269)
	Mitochondrial ribosomal protein S28 isoform 4 (Mitochondrial ribosomal protein S28; isoform CRA_c) (Fragment)
	Metastasis-associated protein MTA1
	NADH dehydrogenase (Ubiquinone) 1 beta subcomplex; 1; 7kDa; isoform CRA_a
	Insulin-like growth factor 2 mRNA-binding protein 3 (IGF2 mRNA-binding protein 3) (IMP-3) (IGF-II mRNA-binding protein 3) (KH domain-containing protein overexpressed in cancer) (hKOC) (VICKZ family member 3)
	TMED5 protein
	Intersectin 1 short form A variant 2
	UDP-N-acetylhexosamine pyrophosphorylase-like protein 1 (EC 2.7.7.-)
	Nuclear factor of activated T-cells; cytoplasmic 4 (NF-ATc4) (NFATc4) (T-cell transcription factor NFAT3) (NF-AT3)
	Signal peptide peptidase-like 2A (Fragment)
vii.	**106 elements included exclusively in "100 µM c-di-AMP+*Pg* LPS↑↑":**
	E3 ubiquitin-protein ligase RNF213

Mycophenolic acid acyl-glucuronide esterase; mitochondrial (EC 3.1.1.93) (Alpha/beta hydrolase domain-containing protein 10) (Abhydrolase domain-containing protein 10)
2';3'-cyclic-nucleotide 3'-phosphodiesterase (CNP) (CNPase) (EC 3.1.4.37)
Cytochrome b-c1 complex subunit Rieske; mitochondrial (EC 7.1.1.8)
39S ribosomal protein L50; mitochondrial (L50mt) (MRP-L50) (Mitochondrial large ribosomal subunit protein mL50)
Mitochondrial Rho GTPase 2 (MIRO-2) (hMiro-2) (EC 3.6.5.-) (Ras homolog gene family member T2)
NADH dehydrogenase [ubiquinone] 1 alpha subcomplex subunit 13 (Cell death regulatory protein GRIM-19) (Complex I-B16.6) (CI-B16.6) (Gene associated with retinoic and interferon-induced mortality 19 protein) (GRIM-19) (Gene associated with retinoic and IFN-induced mortality 19 protein) (NADH-ubiquinone oxidoreductase B16.6 subunit)
Peptidyl-prolyl cis-trans isomerase (PPIase) (EC 5.2.1.8) (Fragment)
cDNA FLJ52100
NADH dehydrogenase [ubiquinone] 1 alpha subcomplex subunit 5
Paraoxonase 2; isoform CRA_a (Serum paraoxonase/arylesterase 2)
Protein canopy homolog 4 (Fragment)
Signal peptidase complex subunit 3 (EC 3.4.-.-)
ATP synthase subunit d; mitochondrial (ATPase subunit d) (ATP synthase peripheral stalk subunit d)
Hydroxysteroid dehydrogenase-like protein 2 (EC 1.-.-.-) (Short chain dehydrogenase/reductase family 13C member 1)
Cytochrome c oxidase subunit 5A; mitochondrial (Cytochrome c oxidase polypeptide Va)
Cdc42 effector protein 3 (Fragment)
Ragulator complex protein LAMTOR5
Mitochondrial NADH-ubiquinone oxidoreductase 75 kDa subunit (NADH dehydrogenase (Ubiquinone) Fe-S protein 1; 75kDa (NADH-coenzyme Q reductase); isoform CRA_b)
Mitochondrial-processing peptidase subunit alpha (Alpha-MPP) (Inactive zinc metalloprotease alpha) (P-55)
Similar to NADH dehydrogenase (Ubiquinone) 1 alpha subcomplex; 9 (39kD) (Fragment)
Acetyltransferase component of pyruvate dehydrogenase complex (EC 2.3.1.12)
Protein dpy-30 homolog (Dpy-30-like protein) (Dpy-30L)
24-kDa subunit of complex I (EC 1.6.5.3) (Fragment)
Barrier to autointegration factor 1; isoform CRA_a
cDNA FLJ58042; highly similar to Protein NipSnap1
Guanine nucleotide-binding protein G(I)/G(S)/G(O) subunit gamma-12
YLP motif-containing protein 1 (Nuclear protein ZAP3) (ZAP113)
Sideroflexin-1
Catalase (EC 1.11.1.6)
Epididymis secretory sperm binding protein Li 134P

cDNA; FLJ94423; highly similar to Homo sapiens mitochondrial ribosomal protein L23 (MRPL23); nuclear gene encoding mitochondrial protein; mRNA
Hepatoma-derived growth factor; related protein 3; isoform CRA_a
Collagen alpha-2(V) chain
Uncharacterized protein
NADH dehydrogenase [ubiquinone] 1 beta subcomplex subunit 10
Dynein light chain roadblock-type 1
V-ral simian leukemia viral oncogene homolog B (Ras related GTP binding protein); isoform CRA_a
MICOS complex subunit
NADH dehydrogenase [ubiquinone] iron-sulfur protein 3; mitochondrial (EC 1.6.99.3) (EC 7.1.1.2) (Complex I-30kD) (CI-30kD) (NADH-ubiquinone oxidoreductase 30 kDa subunit)
NADH dehydrogenase [ubiquinone] flavoprotein 1; mitochondrial (EC 1.6.99.3) (EC 7.1.1.2)
Dihydrolipoyl dehydrogenase (EC 1.8.1.4)
Malate dehydrogenase (EC 1.1.1.37) (Fragment)
Oxysterol-binding protein-related protein 3 (ORP-3) (OSBP-related protein 3)
Sodium/potassium-transporting ATPase subunit beta-3 (Sodium/potassium-dependent ATPase subunit beta-3) (ATPB-3) (CD antigen CD298)
DNA repair protein XRCC1
Dihydrolipoamide S-succinyltransferase (E2 component of 2-oxo-glutarate complex); isoform CRA_a
KIAA1609 protein; isoform CRA_a
cDNA FLJ54557; highly similar to helicase MOV-10
Protein XRP2
Cytochrome b-c1 complex subunit 1; mitochondrial (Complex III subunit 1) (Core protein I) (Ubiquinol-cytochrome-c reductase complex core protein 1)
Alpha-mannosidase (EC 3.2.1.-) (Fragment)
Glutamine amidotransferase-like class 1 domain-containing protein 3A; mitochondrial
Uncharacterized protein DKFZp686E1899
Epididymis secretory sperm binding protein (Peroxiredoxin 3; isoform CRA_b)
Glutathione S-transferase (EC 2.5.1.18) (Fragment)
Elongation factor Tu
Sperm binding protein 1a
Lamina-associated polypeptide 2; isoform alpha (Thymopoietin isoform alpha) (TP alpha) (Thymopoietin-related peptide isoform alpha) (TPRP isoform alpha) [Cleaved into: Thymopoietin (TP) (Splenin); Thymopentin (TP5)]
Nodal modulator 3
Protein VAC14 homolog (Tax1-binding protein 2)
5'-nucleotidase (5'-NT) (EC 3.1.3.5) (Ecto-5'-nucleotidase) (CD antigen CD73)
Peptidyl-prolyl cis-trans isomerase FKBP7 (PPIase FKBP7) (EC 5.2.1.8) (23 kDa FK506-binding protein) (23 kDa FKBP) (FKBP-23) (FK506-binding protein 7) (FKBP-7) (Rotamase)

2-oxoglutarate dehydrogenase; mitochondrial (EC 1.2.4.2) (2-oxoglutarate dehydrogenase complex component E1) (OGDC-E1) (Alpha-ketoglutarate dehydrogenase)
Ectonucleotide pyrophosphatase/phosphodiesterase family member 1 (E-NPP 1) (Membrane component chromosome 6 surface marker 1) (Phosphodiesterase I/nucleotide pyrophosphatase 1) (Plasma-cell membrane glycoprotein PC-1) [Cleaved into: Ectonucleotide pyrophosphatase/phosphodiesterase family member 1; secreted form] [Includes: Alkaline phosphodiesterase I (EC 3.1.4.1); Nucleotide pyrophosphatase (NPPase) (EC 3.6.1.9) (Nucleotide diphosphatase)]
NADH dehydrogenase [ubiquinone] iron-sulfur protein 2; mitochondrial (EC 1.6.99.3) (EC 7.1.1.2) (Complex I-49kD) (CI-49kD) (NADH-ubiquinone oxidoreductase 49 kDa subunit)
D-dopachrome tautomerase (D-dopachrome tautomerase 1) (D-dopachrome tautomerase; isoform CRA_b) (cDNA FLJ76419; highly similar to Homo sapiens D-dopachrome tautomerase (DDT); mRNA)
ATP synthase subunit alpha
Unconventional myosin-VI
Fumarylacetoacetase (EC 3.7.1.2) (Fumarylacetoacetate hydrolase)
Stomatin-like protein 2; mitochondrial (SLP-2) (EPB72-like protein 2) (Paraprotein target 7) (Paratarg-7)
Aconitate hydratase; mitochondrial (Aconitase) (EC 4.2.1.-)
Acid ceramidase
Protein phosphatase 1; regulatory (Inhibitor) subunit 14B; isoform CRA_a
Cytochrome c oxidase subunit 4 isoform 1; mitochondrial (Cytochrome c oxidase polypeptide IV) (Cytochrome c oxidase subunit IV isoform 1) (COX IV-1)
Prohibitin
Prenylcysteine oxidase 1 (EC 1.8.3.5) (Prenylcysteine lyase)
Coiled-coil domain containing 22; isoform CRA_a
KDEL (Lys-Asp-Glu-Leu) containing 2; isoform CRA_a
Glutamate dehydrogenase
Apoptosis-inducing factor 1; mitochondrial (EC 1.1.1.-) (Programmed cell death protein 8)
ATP synthase subunit beta (EC 7.1.2.2)
Adenylate kinase 2; mitochondrial (AK 2) (EC 2.7.4.3) (ATP-AMP transphosphorylase 2) (ATP:AMP phosphotransferase) (Adenylate monophosphate kinase)
Pyridoxal-dependent decarboxylase domain-containing protein 1 (EC 4.1.1.-)
Succinate--CoA ligase [GDP-forming] subunit beta, mitochondrial
Isocitrate dehydrogenase [NADP]; mitochondrial (IDH) (EC 1.1.1.42) (ICD-M) (IDP) (NADP(+)-specific ICDH) (Oxalosuccinate decarboxylase)
Adipocyte plasma membrane-associated protein (Protein BSCv)
Succinate--CoA ligase [ADP-forming] subunit beta; mitochondrial (EC 6.2.1.5) (ATP-specific succinyl-CoA synthetase subunit beta) (A-SCS) (Succinyl-CoA synthetase beta-A chain) (SCS-betaA)
RAB6A protein
cDNA FLJ51907; highly similar to Stress-70 protein; mitochondrial

MICOS complex subunit MIC60 (Cell proliferation-inducing gene 4/52 protein) (Mitochondrial inner membrane protein) (Mitofilin) (p87/89)
Sulfatase modifying factor 2 isoform 2
Succinyl-CoA:3-ketoacid-coenzyme A transferase (EC 2.8.3.5)Protein mono-ADP-ribosyltransferase PARP14 (EC 2.4.2.-) (ADP-ribosyltransferase diphtheria toxin-like 8) (ARTD8) (B aggressive lymphoma protein 2) (Poly [ADP-ribose] polymerase 14) (PARP-14)
Polyglutamine binding protein 1 isoform 1 (Polyglutamine binding protein 1; isoform CRA_c) (Fragment)
Synaptojanin 2 binding protein; isoform CRA_a
cDNA FLJ50890; highly similar to Mitochondrial 39S ribosomal protein L3
Mitochondrial ribosomal protein L14; isoform CRA_b
Cdc42 effector protein 2 (Binder of Rho GTPases 1)
Mitochondrial ribosomal protein L11 isoform a variant (Fragment)
Pleckstrin homology domain containing; family A member 5; isoform CRA_a
RNA binding motif protein 28 isoform 1 (RNA binding motif protein 28; isoform CRA_a)
Epididymis secretory sperm binding protein Li 77p (Fragment)
Thyroid receptor-interacting protein 11 (TR-interacting protein 11) (TRIP-11) (Clonal evolution-related gene on chromosome 14 protein) (Golgi-associated microtubule-binding protein 210) (GMAP-210) (Trip230)
Gap junction protein
Cyclin G-associated kinase variant (Fragment)
cDNA FLJ13562 fis; clone PLACE1008080; highly similar to Homo sapiens hexamethylene bis-acetamide inducible 1 (HEXIM1); mRNA
