# Supplementary material for: Activation of cellular responses by cyclic dinucleotides and porphyromonas gingivalis lipopolysaccharide: a proteomic study on gingival fibroblasts
Source: J Oral Microbiol. 2024 Dec 9;17(1):2431453. doi: 10.1080/20002297.2024.2431453 (PMC11632945; doi:10.1080/20002297.2024.2431453)
Supplement: Supplementary material.pdf [file ZJOM_A_2431453_SM5359.pdf]

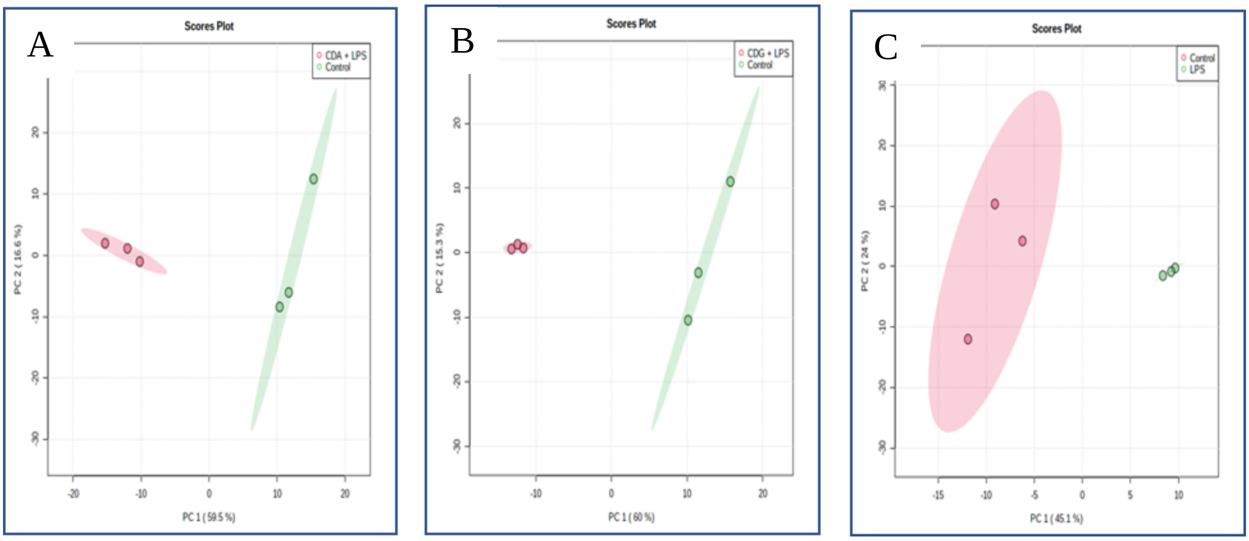

Figure S1**.** Principal component analysis (PCA) showing relation between all significant proteins in gingival fibroblasts following three treatment conditions: (A) c-di-AMP+*Pg* LPS, (B) c-di-GMP+*Pg* LPS, (C) *Pg* LPS. Metaboanalyst software was used to plot the PCA.

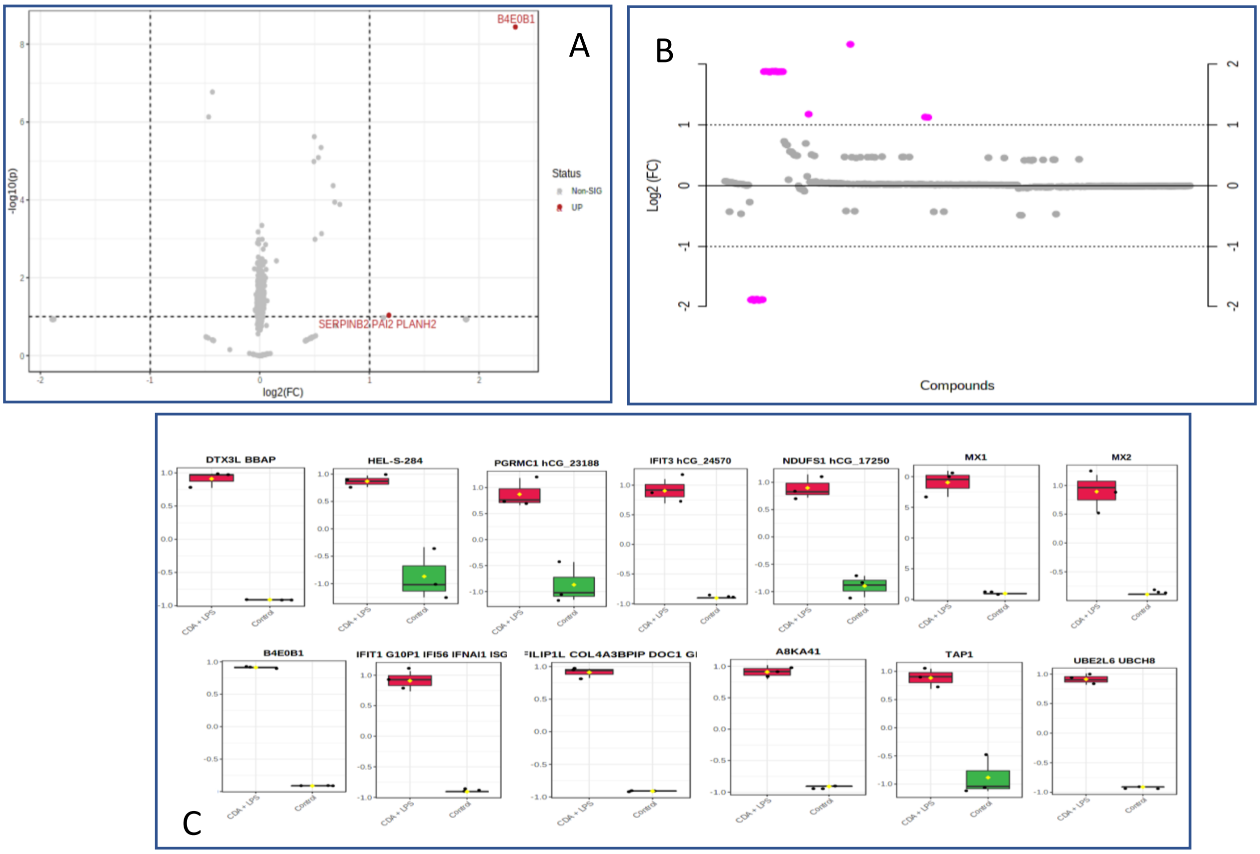

Figure S2: Statistical analysis of C-di-AMP+*Pg* LPS treatment group. (A) Magenta indicates the most affected proteins with Log2 fold change threshold of 1 (2 fold increase). (B) T test with p values transformed by –log10. Showing the smaller p values and the most significant proteins on the above part of the graph (magenta dots). (C) Showing the proteins that represented as magenta dots from figure B.

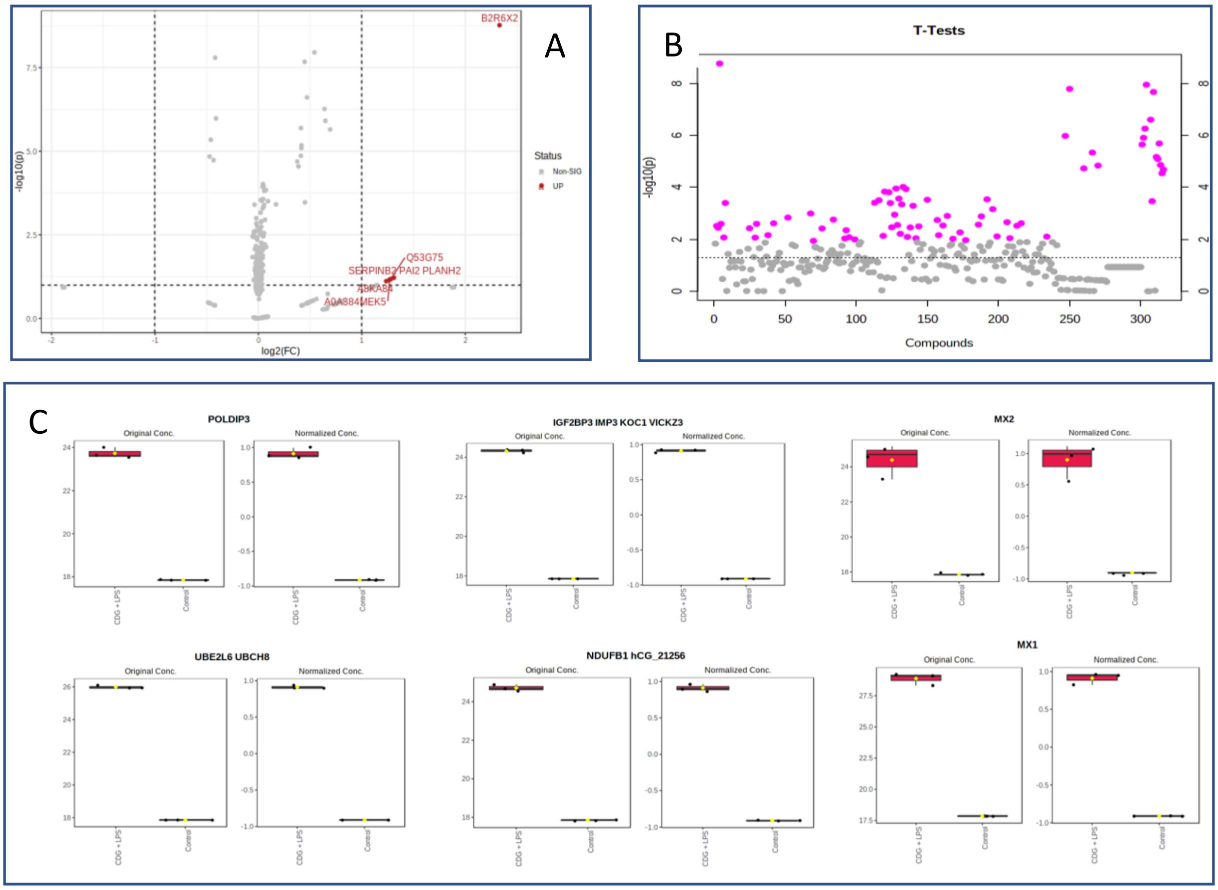

Figure S3: Statistical analysis of c-di-GMP+*Pg* LPS treatment group. (A) Magenta indicates the most affected proteins with Log2 fold change threshold of 1 (2 fold increase). (B) T test with p values transformed by –log10. Showing the smaller p values and the most significant proteins on the above part of the graph (magenta dots). (C) Showing the proteins that represented as magenta dots from figure B.

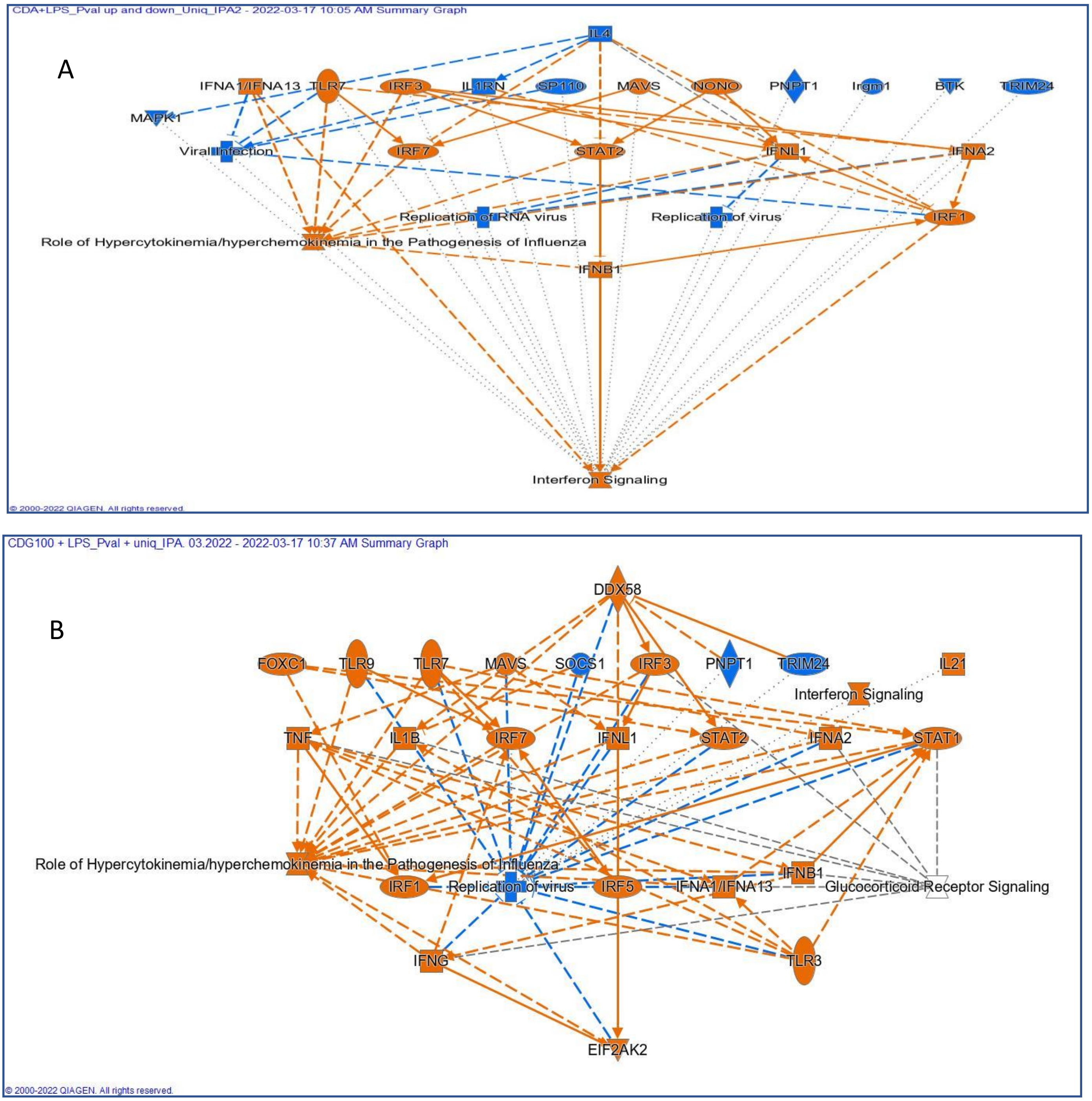

Figure S4. Summary of enrichment analysis of ingenuity pathways regulated by (A) c-di-AMP+*Pg* LPS and (B) c-di-GMP+*Pg* LPS. Orange color (lines or shapes) and blue color (lines or shapes) represented the activation and inhibition respectively, while direct relationships marked as solid lines and indirect relationships by dotted lines. The functional analyses and enrichment were generated using IPA (QIAGEN Inc., https://www.qiagenbioinformatics.com/products/ingenuity-pathway-analysis).

Table S5. Ingenuity pathways exclusively regulated by 100 µM c-di-AMP+*Pg* LPS with -log p > 1.3 ($p < 0.05$).

	**Ingenuity Canonical Pathways**	**-log(p-value)**	**z-score**	**Molecules in c-di-AMP+*Pg* LPS treated fibroblasts**
1	Glutathione Redox Reactions II	1.79	N	PDIA3
2	dTMP De Novo Biosynthesis	1.69	N	DHFR
3	SPINK1 Pancreatic Cancer Pathway	1.6	N	CPD,SMAD3
4	Cell Cycle: G1/S Checkpoint Regulation	1.5	N	GNL3,SMAD3
5	PI3K/AKT Signalling	1.32	N	IL6ST,ITGA2,LIMS1

N= Z-Score was not determined by IPA

Table S6. Ingenuity pathways exclusively regulated by 100 µM c-di-GMP+*Pg* LPS with -log p > 1.3 (p < 0.05).

	**Ingenuity Canonical Pathways**	**-log(p-value)**	**z-score**	**Molecules in c-di-GM+*Pg* LPS P treated fibroblasts**
1	Glucocorticoid Receptor Signalling	2.76	N	CREB1,HLA-B,ICAM1,NDUFA4,NDUFB1,NFATC4,PLAU,POLR2E,SERPINE1
2	Neuroprotective Role of THOP1 in Alzheimer's Disease	2.6	N	APP,CREB1,HLA-B,MME
3	Neuroinflammation Signalling Pathway	2.41	0.816	APP,CREB1,HLA-B,ICAM1,NFATC4,SOD2
4	IL-4 Signalling	2.01	N	HLA-B,HMGA1,NFATC4
5	Tumor Microenvironment Pathway	1.99	1	HLA-B,ICAM1,MMP14,PLAU
6	Coagulation System	1.93	N	PLAU,SERPINE1
7	DNA Methylation and Transcriptional Repression Signalling	1.93	N	MECP2,MTA1
8	Estrogen Receptor Signalling	1.89	1	CREB1,MMP14,NDUFA4,NDUFB1,RBFOX2,SOD2
9	Th1 Pathway	1.7	N	HLA-B,ICAM1,NFATC4
10	Citrulline-Nitric Oxide Cycle	1.63	N	ASL
11	Arginine Biosynthesis IV	1.55	N	ASL
12	Urea Cycle	1.55	N	ASL
13	Pentose Phosphate Pathway (Non-oxidative Branch)	1.55	N	RPE
14	NAD Signalling Pathway	1.46	N	PARP14,POLR2E,SOD2
15	Superoxide Radicals Degradation	1.43	N	SOD2
16	WNT/Ca+ pathway	1.41	N	CREB1,NFATC4
17	Phagosome Maturation	1.4	N	ATP6V0C,HLA-B,VPS28
18	Salvage Pathways of Pyrimidine Deoxyribonucleotides	1.38	N	APOBEC3B
19	Calcium Transport I	1.34	N	ATP2C1
20	Th1 and Th2 Activation Pathway	1.32	N	HLA-B,ICAM1,NFATC4
21	Caveolar-mediated Endocytosis Signalling	1.31	N	HLA-B,ITSN1

22	Hepatic Fibrosis Signalling Pathway	1.31	1.342	CREB1,ICAM1,PDCD4,SERPINE1,SOD2
23	Pentose Phosphate Pathway	1.3	N	RPE

*N= Z-Score was not determined by IPA

Table S7. Ingenuity pathways exclusively regulated by *Pg* LPS with -log p > 1.3 (p < 0.05).

	**Ingenuity Canonical Pathways**	**-log(p-value)**	**z-score**	**Molecules in *Pg* LPS treated fibroblasts**
1	Oxidative Phosphorylation	2.69	N	NDUFAB1,NDUFB9,UQCR10
2	Heme Degradation	2.04	N	HMOX2
3	Induction of Apoptosis by HIV1	2.02	N	SLC25A4,TRADD
4	Telomere Extension by Telomerase	1.47	N	TERF2IP
5	RAN Signalling	1.42	N	KPNA1
6	Necroptosis Signalling Pathway	1.31	N	SLC25A4,TRADD

N= Z-Score was not determined by IPA
